# The Evolution and Role of Molecular Tools in Measuring Diversity and Genomic Selection in Livestock Populations (Traditional and Up-to-Date Insights): A Comprehensive Exploration

**DOI:** 10.3390/vetsci11120627

**Published:** 2024-12-06

**Authors:** Hosameldeen Mohamed Husien, Ahmed A. Saleh, Nada N. A. M. Hassanine, Amr M. A. Rashad, Mahmoud A. Sharaby, Asmaa Z. Mohamed, Heba Abdelhalim, Elsayed E. Hafez, Mohamed Osman Abdalrahem Essa, Saber Y. Adam, Ning Chen, Mengzhi Wang

**Affiliations:** 1 Laboratory of Metabolic Manipulation of Herbivorous Animal Nutrition, College of Animal Science and Technology, Yangzhou University, Yangzhou 225009, China; 008643@yzu.edu.cn; 2 College of Veterinary Medicine, Albutana University, Rufaa 22217, Sudan; dh23045@stu.yzu.edu.cn; 3 College of Animal Science and Technology, Yangzhou University, Yangzhou 225009, China; nedo1.maro2@alexu.edu.eg (N.N.A.M.H.); mh23108@stu.yzu.edu.cn (S.Y.A.); 4 Animal and Fish Production Department, Faculty of Agriculture (Al-Shatby), Alexandria University, Alexandria 11865, Egypt; omr.rashad@alexu.edu.eg (A.M.A.R.); aasni1339@alexu.edu.eg (M.A.S.); 5 Animal and Fish Production Department, Faculty of Agriculture (Saba Basha), Alexandria University, Alexandria 21531, Egypt; asmaazayed@alexu.edu.eg; 6 Animal Production Research Institute, Agriculture Research Centre, Giza 12126, Egypt; heba-sh@alexu.edu.eg; 7 Arid Lands Cultivation Research Institute, City of Scientific Research and Technological Applications, New Borg El Arab, Alexandria 21934, Egypt; ehafez@srtacity.sci.eg; 8 College of Veterinary Medicine, Yangzhou University, Yangzhou 225009, China; 9 State Key-Laboratory of Sheep Genetic Improvement and Healthy-Production, Xinjiang Academy of Agricultural Reclamation Sciences, Shihezi 832000, China

**Keywords:** molecular tools, QTLs, NGS, signatures of selection, GWAS, SNP chip, genomic selection

## Abstract

Distinctive molecular approaches and tools, particularly high-throughput SNP genotyping, have been applied to determine and discover SNPs, potential genes of interest, indicators of evolutionary selection, genetic abnormalities, molecular indicators, and loci associated with quantitative traits (QTLs) in various livestock species. These methods have also been used to obtain whole-genome sequencing (WGS) data, enabling the implementation of genomic selection. Genomic selection allows for selection decisions based on genomic-estimated breeding values (GEBV). The estimation of GEBV relies on the calculation of SNP effects using prediction equations derived from a subset of individuals in the reference population who possess both SNP genotypes and phenotypes for target traits. Compared to traditional methods, modern genomic selection methods offer advantages for sex-limited traits, low heritability traits, late-measured traits, and the potential to increase genetic gain by reducing generation intervals. The current availability of high-density genotyping and next-generation sequencing data allow for genome-wide scans for selection. This investigation provides an overview of the essential role of advanced molecular tools in studying genetic diversity and implementing genomic selection. It also highlights the significance of adaptive selection in light of new high-throughput genomic technologies and the establishment of selective comparisons between different genomes. Moreover, this investigation presents candidate genes and QTLs associated with various traits in different livestock species, such as body conformation, meat production and quality, carcass characteristics and composition, milk yield and composition, fertility, fiber production and characteristics, and disease resistance.

## Simple Summary

This investigation highlights the use of advanced molecular tools, especially high-throughput SNP genotyping, in livestock genetic research. These methods identify SNPs, candidate genes, genetic defects, molecular markers, and QTLs, and facilitate whole-genome sequencing (WGS) and genomic selection, which relies on genomic-estimated breeding values (GEBV). Compared to traditional methods, genomic selection enhances breeding decisions, especially for traits that are sex-limited, have low heritability, are measured late, or need reduced generation intervals. The availability of high-density genotyping and next-generation sequencing supports the argument for genome-wide scans for selection. The investigation emphasizes the importance of these technologies in understanding genetic diversity and adaptive selection, identifies candidate genes and QTLs linked to various traits, and underscores advancements in livestock traits like body conformation, meat quality, milk yield, fertility, fiber production, and disease resistance.

## 1. Introduction

Livestock populations have played a fundamental role in human civilization for millennia, providing essential resources like food, fiber, and labor [1,2]. Throughout history, breeders have strived to improve these populations through selective breeding practices. However, traditional methods often relied on phenotypic observations, leading to slow progress and limited understanding of the underlying genetic mechanisms [3,4].

Traditionally, phenotypic traits have been used as indicators to study biodiversity both within and among breeds for many years. Various studies have utilized phenotypic markers such as blood protein polymorphism, isozyme variability (Table 1), and blood plasma to measure genetic variation, genetic distances, heterozygosity, and genetic structure. This knowledge has aided animal breeders in conducting genetic improvement programs and selection efforts [5].

The recent emergence of molecular tools has revolutionized the field of livestock breeding, offering unprecedented insights into genetic diversity and selection strategies [6,7]. This introduction explores the evolution of these tools, their impact on measuring diversity within livestock populations, and their role in guiding genomic selection for enhanced performance and sustainable practices [8,9].

For centuries, breeders relied on visual assessment of phenotypic traits, such as growth rate, milk production, or wool quality, to select breeding stock [10,11]. Additionally, pedigree information played a crucial role in these practices, allowing for the tracking of ancestry and the selection of animals expected to pass on desirable traits [12]. However, these methods faced several limitations:

Phenotypic evaluation can be subjective and is influenced by environmental factors such as (a) limited information: phenotypes reflect the combined effects of numerous genes and environmental influences, making it difficult to isolate the genetic basis of desired traits [13,14] and (b) slow progress: traditional methods led to gradual improvement over generations, and the genetic basis for desirable traits remained largely unknown [15,16].

The advent of molecular tools provided a more objective and detailed picture of genetic variation within and between breeds. These markers directly assess the genetic makeup of an animal, offering several advantages [17,18].

Molecular markers are based on the animal’s DNA, providing an objective measure of genetic variation [19]. A wider range of genetic information can be analyzed, revealing hidden patterns of diversity and uncovering the underlying genetic architecture of complex traits. This shift towards molecular tools paved the way for further advancements in understanding livestock genomes [20,21]. The power of NGS technologies has opened entirely new avenues for exploring livestock genomes [22,23]. NGS allows for the rapid and cost-effective sequencing of entire genomes, enabling researchers to (a) identify candidate genes: locate genes associated with desirable traits through genome-wide association studies (GWAS) [24,25], (b) predict breeding values: develop more accurate estimates of an animal’s genetic potential for passing on beneficial traits to offspring [26], and (c) personalized selection: tailor breeding programs to individual animals based on their unique genetic makeup [27,28]. These advancements hold immense potential for revolutionizing livestock breeding by enabling the selection of animals with superior genetic potential, ultimately leading to (a) improved production efficiency: increased milk [29,30] and meat production [31], faster growth rates [30,32], and enhanced fiber quality [33,34], (b) enhanced disease resistance: selection of animals with genetic resistance to common diseases, reducing reliance on antibiotics and improving animal well-being, and (c) sustainable practices: developing breeding programs that optimize resource utilization and minimize environmental impact. Furthermore, these tools can inform conservation efforts by identifying and preserving valuable genetic diversity within endangered livestock breeds [35,36].

By leveraging the power of molecular tools, we can build a more sustainable future for livestock breeding, ensuring the continued success of this critical sector for generations to come [37,38,39].

The objectives of this investigation were as follows: (1) Survey the evolution and impact of molecular tools in livestock genetic diversity and genomic selection. (2) Analyze the advancements in high-throughput SNP genotyping and whole-genome sequencing (WGS) in the context of livestock populations. (3) Evaluate the role of next-generation sequencing (NGS) in identifying candidate genes and quantitative traits (QTLs) associated with key traits in livestock. (4) Compare modern genomic techniques with traditional approaches in livestock genomics. (5) Discuss the implications of personalized breeding programs for livestock production efficiency and conservation.

## 2. Materials and Methods (Methodology)

Over 343 sources, encompassing a range of published research papers, scientific journals, and international books, as well as master’s and doctoral theses, have been referenced. These works were disseminated by prominent publishers such as Elsevier, Springer, ScienceDirect, Wiley, MDPI, Taylor & Francis, IntechOpen, IEEE Xplore Digital Library, JSTOR, PubMed, Nature Research Journals, Oxford Academic, and other platforms. Comprehensive investigations were conducted on these platforms to gather extensive knowledge about livestock.

Furthermore, the global databases relevant to the genomic databases and the animal genetics, production, and breeding sectors were used [40,41], as shown in Supplementary File S1.

## 3. Animal Genetic Resources (AnGR) Biodiversity

Given the diminishing diversity in animals, there has been an increased focus on studying the variation both among and within animal genetic resources. In livestock, animal genetic resource diversity manifests both within and between breeds. This diversity is crucial for planning and implementing genetic improvement programs [42,43,44]. Quantifying genetic variation requires more than just morphological studies; molecular techniques provide a more reliable measure and are now extensively used to assess biodiversity among populations [42].

Molecular genetics offers a deeper understanding of how genetic variation is distributed within and between livestock breeds, playing an essential role in animal breeding programs and genetic improvement strategies [43,45]. At the molecular level, breed and population structures can be clarified using highly variable loci, which provide extensive information on individual genotypes [42].

## 4. Developments in Estimating Animal Genetic Values

Since the dawn of domestication, farmers and breeders have aimed to identify target traits to pass on to future generations. Utilizing pedigree information or phenotype measurements, they have made decisions about which farm animals to retain and mate. These decisions have been pivotal in effecting necessary changes in the characteristics of farm animals, enhancing their economic efficiency. In recent decades, advances in genetic techniques and sophisticated statistical analyses have enabled breeders to estimate the genetic values (GV) of their herds, drawing on pedigree information, phenotypes, and genotypes [46,47]. For instance, the global application of mixed model procedures to achieve the best linear unbiased prediction (BLUP) of estimated breeding values (EBVs) has been instrumental in evaluating GV across all commercial livestock industries. Numerous studies have demonstrated that using EBVs to guide mating and selection decisions has significantly contributed to genetic progress and, consequently, to the profitability of raising animals [46,47].

Since 2007, the advent of NGS has made it possible to sequence the genomes [48] of goats (*Capra hircus*) [49], sheep (*Ovis aries*) [50], cattle (*Bos tatrus*) [51], and buffaloes [52], (Table 2). This progress has facilitated the development of high-density SNP chips such as the Goat SNP K50 Bead-Chip, which includes 53,347 SNPs [53], and the Ovine 600K SNP chip, comprising 606,006 SNPs. The availability of such advanced technologies has enabled the use of genome-wide data in animal breeding improvement programs.

**Figure 1:**
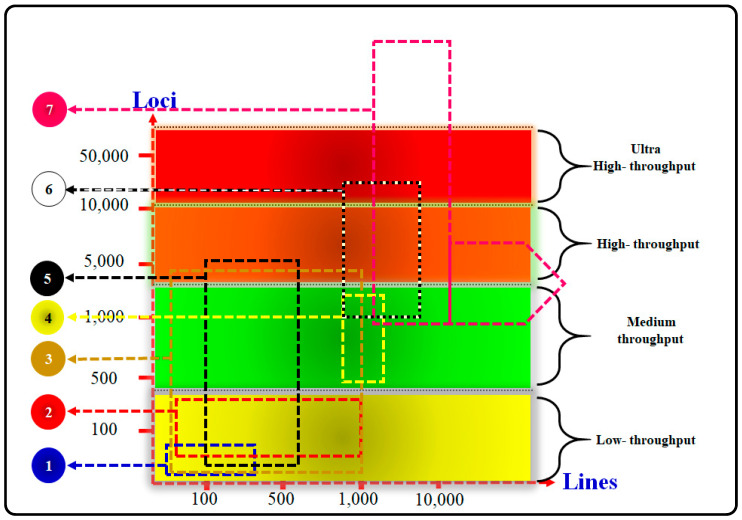
Marker assay platforms for genotyping, from low to ultra-high-throughput; (1) SSRs, (2) BeadXpress, (3) KASPar assay, (4) Golden-Gate assay, (5) DArT assay, (6) Infinium assay, and (7) genotyping by sequencing: **simple sequence repeats (SSRs) assay**, also known by various names such as microsatellites, short tandem repeats, variable number tandem repeats, simple sequence length polymorphisms, and sequence-tagged microsatellite analysis, is a pivotal technique in genetic studies. These microsatellites consist of repetitive DNA segments with motifs ranging from one to six or more base pairs, typically repeated up to 50 times or more. These sequences are interspersed throughout an organism’s genome, both in coding and non-coding regions, and exhibit higher mutation rates compared to other DNA areas. This mutability results in significant genetic diversity, making them ideal markers in various genetic applications, including population genetics, breeding programs, and conservation biology. **Microsatellite techniques** are employed across diverse studies using both low- and high-throughput genotyping methods. Simple sequence repeats involve various techniques such as sequence-tagged microsatellite site, fluorescent in-situ hybridization of chromosome oriented, retrotransposon-microsatellite amplified polymorphism, selective amplification of microsatellite polymorphic loci, multiple primer polymerase chain reaction/inter-simple sequence repeat, random amplified microsatellite polymorphisms, degenerate random amplified polymorphic DNA, recombinant random amplified microsatellite polymorphisms, random amplification of hypervariable motifs, and random amplification of microsatellite polymorphic DNA, each offering specific advantages for identifying and analyzing microsatellites. **BeadXpress assay** integrates VeraCode digital microbead technology with dual-color laser detection, allowing efficient single nucleotide polymorphism scanning across many individuals using the BeadXpress Reader System. This assay provides high-quality data and flexibility through dual readings of the beads’ code and fluorescence intensity signals, making it ideal for applications needing precision and sensitivity, such as pharmacogenomics and genetic association studies. **Kompetitive allele specific polymerase chain reaction assay** is a fluorescence-based genotyping method derived from the polymerase chain reaction, employing allele-specific oligonucleotide extension and fluorescence resonance energy transfer for signal creation, offering cost-effective and efficient genotyping. **Golden-Gate assay** is a protocol for high-quality, high-multiplex genotyping, enabling extensive loci multiplexing through advanced chips like a 1536-plex single nucleotide polymorphism marker chip, supporting robust genomic investigations. **Diversity Arrays Technology assay** serves as a high-throughput, genome-spanning method suitable for polyploid analysis by employing complexity reduction and DNA hybridization-based methodologies to assay thousands of markers. **Infinium assay** stands out as an ultra-high-throughput single nucleotide polymorphism genotyping system capable of identifying and scoring up to 2.5 million single nucleotide polymorphisms per DNA sample through the direct hybridization of genomic targets to array-bound sequences. This platform’s high single nucleotide polymorphism analysis capability is favored in comprehensive studies like whole-genome association and population genetics due to its ease of use and data analysis. Lastly, **Genotyping-by-sequencing** is a pioneering next-generation sequencing method for discovering and genotyping single nucleotide polymorphisms in animal genomes, utilizing a streamlined process involving restriction enzymes, barcode adapters, polymerase chain reaction amplification, and sequencing, which is proving effective for genome-wide association studies, genetic diversity research, molecular marker discovery, and large-scale breeding programs.

Molecular tools have revolutionized animal breeding worldwide by accelerating genetic gains compared to traditional methods (Figure 1). Improvements have been achieved in various livestock products, such as beef and milk from cattle, using both traditional and advanced technological methods [42,87,88].

## 5. The Potential of Genetic Diversity in Livestock Populations

**Figure 2:**
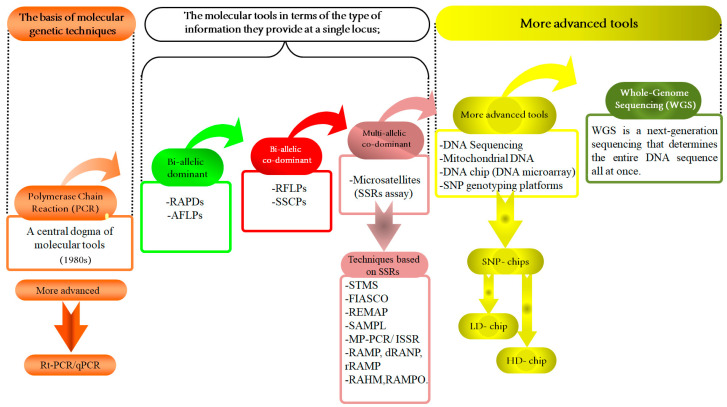
Molecular tools and their applications in animal genetic studies: a summary of development. This figure presents a comprehensive overview of the evolution and application of various molecular tools in animal genetics, framed within the central dogma of molecular biology. At the core is polymerase chain reaction (PCR), a pivotal technique that has revolutionized genetics by enabling the amplification of specific DNA sequences. Building upon this foundation, the Figure explores bi-allelic techniques, such as RAPDs and AFLPs approaches, then bi-allelic co-dominants, such as RFLPs and SSCPs, and co-dominance principles such as SSRs to assess genetic variation and allow for the detection of both alleles in a heterozygote, providing a detailed genetic profile. This further expands to multi-allelic approaches, incorporating advanced sequencing technologies like DNA sequencing, SNP chips, mitochondrial DNA (mt-DNA) analysis, and whole genome sequencing (WGS). These tools facilitate comprehensive assessments of genetic diversity and evolutionary relationships across animal species. The integration of high-density (H.D.) and low-density (L.D.) SNP chips has become instrumental in large-scale genetic studies. Also, it illustrates the application of these molecular tools in genomic selection (G.S.). By utilizing the detailed genomic information obtained from these technologies, G.S. enables the selection of individuals with superior genetic traits, thus optimizing breeding programs. This integration of advanced molecular techniques underscores the transformative potential of genomic selection in enhancing productive and health-related traits in animals, contributing significantly to the advancement of animal genetic research and breeding efficiency.

The assessment and identification of genetic resources within and among indigenous livestock populations serve as effective tools for the swift enhancement of economically valuable traits by leveraging WGS variations. That became possible by applying new technologies of sequencing [89], (Figure 1 and Figure 2). For precise determination and characterization of genetic resources, accessing diverse WGS data are crucial. This access aids in identifying genes and allele mutations linked to various environmental conditions, particularly those that play a critical adaptive role [90].

Obtaining genome-enhanced breeding values (GEnBV) at an early age to select young individuals before extensive progeny data are available has had significant impacts on breeding programs across numerous species [91]. Though the selection programs that depended on the EBVs obtained from phenotypes have been very useful and successful, they encounter several limitations and known issues due to relying solely on phenotypes for selection: (1) the high cost and additional time required to record phenotypic trait values, (2) the restriction of some target traits to females only, such as milk yield, (3) the extended period needed to measure certain traits, such as longevity, and (4) other traits, such as meat quality, need the slaughtering of individuals. (5) Resistance traits require the animal to get sick when exposed to the disease in order to measure disease resistance [92]. All these and many other obstacles limit genetic progress.

Animal breeders have accumulated substantial experience in exploring opportunities and developing decision-making systems for obtaining early measurements of target traits in selection candidates that can enhance the accuracy of estimated breeding value (EBV) estimation at a young age. One notable early application involved using blood groups as markers for disease resistance in chicken selection [93]. Historically, employing molecular genetics tools for quantitative trait selection was prohibitively expensive [94].

Nevertheless, the advent of new molecular tools and techniques, such as high-density SNP panels [95] and WGS [90] (Figure 2), has positively impacted livestock genetic improvement. Furthermore, genomic evaluation methods can significantly increase the accuracy and effectiveness of GEBV estimation, thereby accelerating the selection response. Although the accuracy of methodologies utilizing genomic prediction, like genomic best linear unbiased prediction (GBLUP) and BLUP-SNP, is constrained in smaller families or populations [96], GBLUP has demonstrated the increased accuracy of EBVs compared to pedigree-based BLUP [48].

Therefore, this investigation explores the impact of molecular tools on livestock development. It delves into key topics for applying genomic data, reviews influential candidate/potential genes, selection signatures (SS), and QTLs, and underscores the importance of combining phenotypic traits with genomic insights in genomic selection (GS). Recent advancements in animal genomics are also presented.

## 6. Advanced Molecular Techniques for Assessing Biodiversity

**Figure 3:**
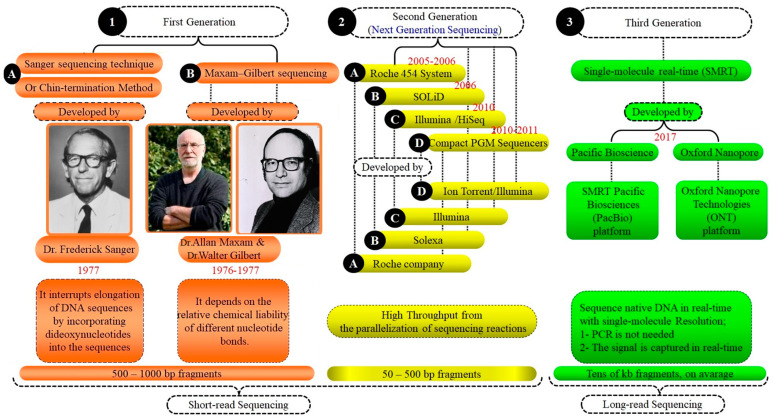
Comparison of generation sequencing systems: a summary of the development. This Figure highlights the advancements through three generations of sequencing systems. First generation: Sanger and Maxam–Gilbert methods from the 1970s introduced basic DNA sequencing. Second generation: next-generation sequencing (NGS) increased speed and reduced costs with high-throughput capabilities. Third generation: single-molecule real-time (SMRT) sequencing offers longer reads and deep genomic insights. Each generation enhanced our ability to decode and understand genetic information.

Over the past 50 years, advancements in molecular techniques have greatly enhanced the study of biodiversity in animal genetics (Table 3 and Figure 3). Key molecular methods include RAPD, AFLP, RFLP, SSCP, MST/SSRs (Figure 2), mitochondrial DNA (mt-DNA), DNA microarrays (biochips/DNA chips), and both low- and high-density SNP chips, as well as WGS (Figure 3). These tools are instrumental for animal breeding research and applications [42,93].

Significantly, emerging ideas in the domain of animal genetics and breeding underscore the importance and urgency of identifying and characterizing new polymorphisms linked to critical traits. Extensive research has already been conducted, with ongoing efforts continuing in order to revamp genomic maps. This work aims to elucidate the impact of allelic variations on linkage analysis and quantitative phenotypes, thereby expediting genetic enhancement through various molecular techniques within breeding initiatives. Utilizing prolific varieties to categorize and map genes has been considered a principal approach by geneticists. Similarly, the identification of many famous genes that are accountable for several traits in livestock species has been essential. Technologies like cloning and transgenics to manipulate genotypes have been actively employed in recent years. However, researchers should critically analyze the viability of these approaches [42,87,113]. The following are some advanced molecular tools/methods for GS and measuring biodiversity.

### 6.1. The High-Density of SNP Genotyping

SNPs/snips are the latest contribution to investigating DNA sequence variation. They are discovered where various nucleotides occur in the same position across the DNA sequence in both non-coding and coding regions of the genome at a rate of “one SNP” per thousand base pairs (bp) [114,115].

There are three generations of sequence systems (GSS). (1) The first generation, including the Sanger sequencing technique (Chin-termination method) and Maxam–Gilbert sequencing, started in the 1970s. (2) The second generation (NGS). (3) The third generation (single-molecule real-time SMRT) (Figure 3). The evolution of genetic tools/approaches through the last few decades is shown in (Figure 2 and Figure 3).

#### 6.1.1. Maxam and Gilbert Sequencing

It is a tool/method of sequencing developed and modified by Allan Maxam and Walter Gilbert (1976–1977). This approach depends on nucleobase-specific partial chemical modification of DNA and subsequent cleavage of DNA’s backbone at sites adjacent to the modified nucleotides [116], (Figure 3).

#### 6.1.2. Next-Generation Sequencing (NGS) “SNP Chips and Genotyping”

**Figure 4:**
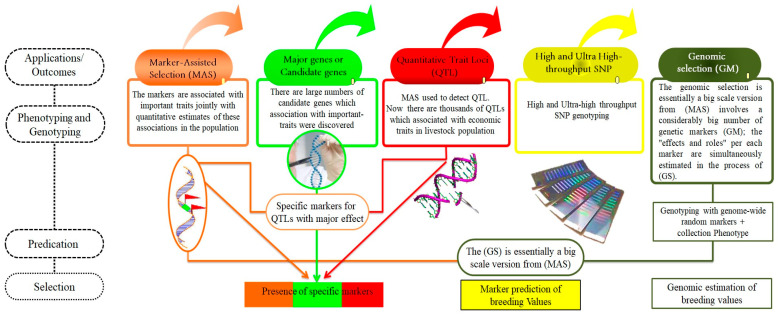
The applications/outcomes of advanced molecular tools: MAS, major genes, QTL, high-density of SNP genotyping, and genomic selection. This Figure illustrates the impact of advanced molecular tools in animal genetics, demonstrating how they enhance breeding and selection processes. Key applications include MAS, which uses molecular markers to improve the accuracy of trait selection and genetic efficiency. The identification of major genes provides insights into genes with significant effects on traits, enabling targeted genetic improvements. QTL mapping identifies genomic regions affecting economically important traits, guiding focused breeding strategies. High-density SNP genotyping facilitates a detailed genome analysis, enhancing our understanding of genetic diversity and adaptability. Additionally, genomic selection leverages genomic information to select individuals with superior genetic potential, thereby accelerating genetic gains in breeding programs. Collectively, these tools empower the optimization of productive and health-related traits in animals through sophisticated genetic applications.

Numerous studies have been conducted on molecular markers, notably SNP panels, to devise conservation strategies and assess genetic diversity [117]. The current availability of SNP panels has simplified the examination of genomic diversity in animals. These panels have largely supplanted microsatellites for detecting genetic diversity and establishing parentage in numerous species. Among the various types of genetic markers, SNP panels are frequently employed due to their effectiveness [118], (Figure 2, Figure 3 and Figure 4). A total of >4 million SNPs in the human genome were genotyped through the second phase of the Hap-Map project [119]. The SNPs that have been discovered in dogs approaches >2.5 million [120], in chickens > 2.8 million [121], in mice > 8.2 million [122], and in cattle > 60,000 [123]. In humans, genome-wide association studies (GWAS) have used these markers to discover genomic regions or sequence variants associated with 40 complex diseases [124].

In animals, the development of the NGS permitted checking of the sequencing of buffaloes [52], cattle [51], sheep [50], goats [49], deer [125], pigs [126], and chickens [127]. Worth mentioning, the genomic studies in sheep were first possible in 2009 utilizing the 50K ovine SNP chip (Table 2). The high-density SNP data can provide chances to discover QTLS and to build the genetic architecture of important quantitative traits [92,128].

The initial 50K bovine SNP chip, containing over fifty thousand SNPs, became available in 2011 and was developed using ten geographically and biologically diverse breeds. Conversely, the International Goat Genome Consortium, established in 2012, aimed to enhance genomic tools for goat genetics [129]. By 2013, the consortium had collaborated to create the 52K SNP chip [95], known commercially as Illumina, by integrating genome libraries and WGS from eight Asian and European breeds [129]. Dong et al. [49] released the first goat genome assembly. The identification of twelve million SNPs facilitated the creation of 53K and 54K SNP chips, which have gained widespread global usage [95]. A 54K chip has also been utilized to analyze the genomes of cattle and buffaloes [130]. More recently, 60K and 62K SNP chips have been introduced [131].

A commercial platform for different animal species is available to scan the genotyping of hundreds to thousands of SNPs through the whole genome at a reasonable cost (about $150/individual), depending on the volume of animals. Panels that have over seven hundreds-k of SNP have become available for cattle [132]. The recent availability of high sequencing technologies led to the production of SNP chips. Hundreds of thousands of beef bulls, dairy cows, sheep, and goats have been checked and genotyped utilizing these platforms. Presently, for most livestock species like cattle, buffalo, sheep, goats, horses, poultry, and pigs, chips with forty to sixty-five thousand SNPs are accessible. However, for some other species, these SNP chips remain in the developmental stage [92].

#### 6.1.3. Third-Generation “Single-Molecule Real-Time (SMRT)”

The second version of the genome that exploits single-molecule with long-read sequencing was released in 2017 by (Pac.Bio) [133]. In this version, the gene annotation has been improved considerably and the positions of SNPs on the chips have been modified and updated [134], (Figure 3).

It is capable of discovering SNPs and represents one of the most interesting approaches in genotyping because it is amenable to high- and ultra-high-throughput automated analysis. Recently, this approach is under active development [135]. It works by reading the nucleotide sequences at the single-molecule level, unlike existing methods that require breaking long DNA strands into small segments and then inferring nucleotide sequences by amplification and synthesizes [136].

Through the application of appropriate statistical methods, it offers a robust approach for the direct discovery and identification of traits linked to the sequence variations that underlie the molecular mechanisms of adaptation and domestication [137].

## 7. Outcomes of Utilizing Molecular Tools in Animal Breeding

### 7.1. Marker-Assisted Selection (MAS)

One of the most important outcomes of utilizing molecular tools is to identify the markers used in animal selection. In general, molecular markers (MM) are fragments of DNA that are associated with certain locations within the genome. They are utilized to identify a particular DNA sequence in a pool of unknown DNA [113,114]. The marker-assisted selection (MAS), also known as marker-aided selection, is an indirect selection process where a target trait is selected based on a marker (DNA/RNA, biochemical, or morphological variation) linked to a target trait (e.g., productivity, reproduction, stress tolerance, disease resistance, etc.) (Figure 4). The concept of MAS was originally found as early, using both genotypic and phenotypic information. In addition to the preceding applications, MAS provides a definitive test of ancestry [138] and ensures that a given breed or individual carries a particularly rare or useful gene included in the conservation program [139]. Utilization of MAS depends on the relationship between the quantitative traits and MM (Figure 4). It helps to conquer the restrictions of traditional selection and can be utilized on sex-limited and low-heritable traits, and those measured later, towards the end of an animal’s life. MAS increases the genetic gain by reducing the generation interval but requires information on alleles or markers associated with the target traits jointly with quantitative estimates of these associations prior to application for a specific animal population [140].

### 7.2. The Global Usefulness of High-Throughput SNP (SNP Panels)

The Adapt Map project gathered 53K genotypes from 140 breeds across 17 countries, aiming to select a high-performance SNP panel for parentage and assignment in 91 populations [118]. These SNPs were validated on Alpine and French Saanen goats. The Caprine-SNP50k Bead-Chip was used to study Swiss goat breeds and identify regions affecting milk composition, growth, and coat color [118]. The Goat-50-K-SNP Bead-Chip also helped pinpoint selected genomic regions and showed significant molecular changes in small ruminant species [141]. Commercial 50K SNP chips, available for about six years [95], have been used nationally, regionally, and continentally to explore goat diversity [134]. Similar SNP advances in sheep, cattle, pigs, and chickens have identified genes linked to positive selection and phenotypic variation [42]. Brito et al. [142] found that 1151 individuals from 9 breeds genotyped with the 50K Bead-Chip showed genes related to economic traits, including milk protein, reproduction, feed efficiency, body mass, conformation, fat metabolism, and heat tolerance [42,94].

In the study by Ren et al. [143], who examined the GWA on 24 phenotypes of the Chinese two-horned Sishui Fur breed and 22 phenotypes of the four-horned breed utilizing the Ovine SNP Bead-Chip and found a genomic region on chromosome 2, involving 132.0–133.1 Mb that included the top ten SNPs, four of them were significant and five highly significant haplotypes related to the polycerate phenotype. It is worth mentioning that in mice and humans, that region involves the *HOXD*, *KIAA1715*, and *EVX2* genes, which have an association with the formation of genital buds and limbs. Colli et al. [144] utilized the Goat SNP 50 Bead-Chip on more than four thousand individuals of goats representing 169 breeds from 35 countries across 6 continents and detected several candidate genes and their relationship to some important traits; the results also indicated that the extensive genes flow was within the following well-defined geographical regions: Ethiopia–Cameroon, Mali–Burkina Faso–Nigeria, Morocco, Pakistan, and Southern Europe, and was also between some Zimbabwe breeds and Turkish breeds as well as between the breeds from North Africa and southern Europe. Furthermore, there was a marked lack of genes flow in the wild insular goat breeds from Ireland, the Canary Islands, Iceland, Malawi, Mozambique, Madagascar, Pakistan, and Iran.

Qiao et al. [145] suggested the design of an SNP chip for solution hybrid selection (SHS) depending on the strategy of target enrichment, thus, a 66K SNP chip was created for the Cashmere goat from the data for WGS relating to 73 heads. The comparison of the 66K SNP chip with the available commercial SNP chip showed that the depth of the target for SNPs was 40× and the testing of this chip with the data for WGS for 436 heads showed that the call rates of the SNP were between 95.3 and 99.8%. The capture for the SNP’s target flank regions appeared to be 200 bp when the chip was tested by GWAS analysis for the fineness of Cashmere hair using fiber diameter. The results showed that using the 66K SNP chip is useful and can be applied to many other species.

### 7.3. Exploring the Mitochondrial DNA Sequence

NGS has been utilized to identify mitochondrial DNA (mt-DNA) variants [146]. Due to its rapid evolution rate, strict maternal inheritance, and lack of recombination, mt-DNA is highly informative for examining diversity among closely related livestock and individual animals across species [147]. Moreover, mt-DNA remains a crucial tool in molecular phylogenetics and population genetics research [148].

Variations or mutations in mt-DNA can significantly impact the efficiency of oxidative phosphorylation, thereby influencing cellular energy production and, consequently, animal performance [149]. Molecular techniques have allowed for the identification of these mt-DNA variations and their associations with performance traits in various livestock species; Mannen et al. [150] found an association between carcass characteristics and Mt-DNA variation in Japanese black steers (cattle). Also, Smith et al. [149] reported that low male fertility was associated with a unique mt-DNA haplotype in brown hares (Lepus). In addition, the mt-DNA investigations contributed strongly to understanding the role of the different mitochondrial haplotypes in our understanding of the nature of phenotypic traits and predisposed individuals and protecting them against several diseases. Also, it detected the association between fertility and twinning rates in pigs [151], time to pregnancy, milk, and meat quality in Holstein cows [152], and longevity [153] and tolerance to heat [154] in both humans and animals.

### 7.4. Identification of Potential Genes Associated with Economic Traits in Livestock

Numerous candidate genes associated with critical traits have been uncovered and identified across various species through the use of molecular tools [155]. Several investigations reported that there are hundreds of genes discovered coincidently with the release of the reference of the genome sequence in the different animal species (Tables S1–S8). The association between candidate genes and many important traits, such as production and reproduction traits and disease, involves a lot of genes; the effects of those candidate genes vary as follows: (1) part of them affect the metabolism, physiological pathways, and expression of polymorphic phenotypes such as *GH*, *GHR*, *IGF-I*, *CAPN-1*, *CAST*, *POU1F1*, *LEP*, *MSTN*, and *IGFBP-3* gene, which are important for growth traits, birth and weaning weights, muscle growth, bone formation, body conditions, body size, meat quality, and meat production (Tables S1–S3). (2) Another part affects fertility and reproduction, such as *MTNR1A*, *FOXL2*, *AMEL*, *SRY*, *BMP-15*, *GDF-9*, and *BMPR-1B* genes, which are necessary for fertility, infertility, proliferation, and sex determination (Table S4). (3) The third part of genes are candidates for milk composition and milk yield traits, such as the family of casein genes (Table S5). Genes associated with casein production in milk protein are already utilized in breeding programs (Table S5). (4) Key genes influencing fiber traits, such as *FGF-5*, *IGFBP-7*, *MC1R*, and *KAP*, are critical for characteristics like hair length, color, and type (Tables S6 and S7). (5) Additionally, genes relating to the immune response and disease resistance include *MHC-DRB3* and *-DQA2* [156], Tmem-154 for MAEDI-VISNA resistance [157], *Prp* for scrapie resistance [157], and *Socs-2* for mastitis susceptibility [158]. Notably, *Fec-L* and *Prp* genes in global sheep populations, and the α-s1-casein gene in French goats, are used for pre-selecting progeny test candidates [159].

Also, the utilization of advanced molecular tools resulted in mapping many useful individual genes in small ruminants [160] and dairy and beef cattle [161]. Also, veterinary tests utilized advanced molecular tools for the diagnosis of genetic diseases such as deficiency of uridine-monophosphate-synthase (DUMPS), complex-vertebral-malformation (CVM), and bovine leukocyte-adhesion-deficiency (BLAD) in cattle that are tested to find out if the seed stock bulls are either carriers or non-carriers of these autosomal recessive mutations [162,163]. Small ruminants’ testing for the Prp gene associated with scrapie has been identified by molecular tools [42].

### 7.5. Detection of QTLs

**Figure 5:**
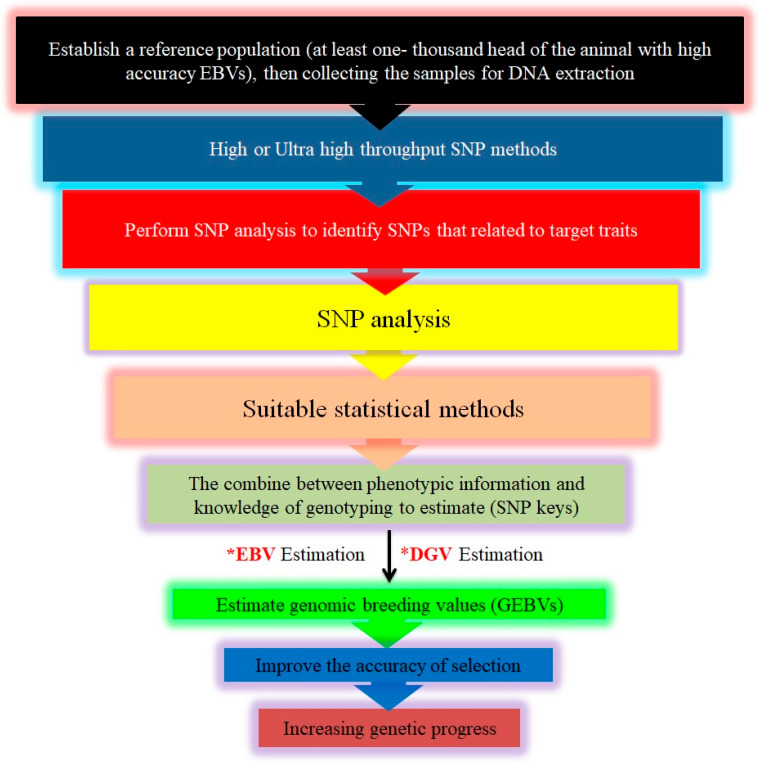
Steps in implementing genomic selection (GS). This Figure outlines the comprehensive process for implementing genomic selection in livestock breeding. It begins with the collection of phenotypic and genotypic data from a reference population utilizing high-throughput SNP genotyping technologies. Next, the effects of individual SNPs on various traits are estimated using advanced statistical models. These effects are used to calculate direct genomic values (*DGV), which represent each individual’s genetic potential based solely on their genomic information. The DGV are then integrated with traditional estimated breeding values (*EBVs) to produce genomic-estimated breeding values (GEBV), offering a more accurate prediction of an individual’s breeding potential by combining genomic data with pedigree information. Superior individuals, identified through their GEBV, are selected as breeding candidates, allowing more efficient improvement of desired traits. Finally, these selected individuals are used in breeding programs to enhance trait optimization and accelerate genetic gain across the livestock populations.

In the previous molecular applications, the gene associated with the target trait may have been either a single one with large effects (major gene) [87], such as those affecting coat color, polledness, and double muscling, or could be one of many genes affecting the quantitative trait of interest (minor gene), such as those associated with milk yield, growth, or wool production or quality [88]. The loci that affect a quantitative trait are termed QTLS or economic trait loci (ETL). By another meaning, QTLs are hypotheses that specific regions on a chromosome contain several genes that make a critical contribution to the expression of a quantitative/complex trait. In populations that had effective improvement programs for many generations, MAS most likely will be for QTLS rather than for major genes, since major genes with large favorable effects are likely to have been fixed in this population already [138], (Figure 5). QTL analysis started in the nineties and now several QTLs for many animal species are available (Table 4) followed by many sequences, as described in Table 2, with the help of different molecular tools.

It was estimated that using markers linked to QTLs in dairy and other livestock breeding programs could increase animal response by up to 30% [164]. Whether there are benefits from MAS, and the scale of such potential benefits, will depend on the QTL effect, the strength of the linkage between the marker and the QTL, and the rates of possible changes by conventional means [165]. The utilization of modern molecular tools led to the discovery of thousands of QTLs associated with economic traits in livestock species, as shown in Table S8. Table S8 summaries most of the important economic traits of interest to breeders and producers (carcass characteristics, carcass composition, bone characteristics, body conformation, growth traits, fiber traits, milk traits, and resistance to diseases) working in animal production and their association with different QTLs and their sites in most farm animals (beef cattle, dairy cattle, meat sheep, meat goat, dairy goat, hair goat, cashmere goat, etc.), with a mention of the specific breeds.

**Table 4:** Number of QTLs for many species based on animal QTL database, updated to 2023/2024.

No.	Species	No. QTLs	Publications No.	Traits	Ref.
1	Goat	128	6	Represent 25 different traits	[166,167,168,169,170]
2	Chicken	16,656	376	Represent 370 different traits	
3	Sheep	4416	226	Represent 266 different traits	
4	Cattle	193,216	1111	Represent 684 different traits	
5	Horse	2636	106	Represent 65 different traits	
6	Pig	35,846	773	Represent 693 different traits	

### 7.6. Obtaining the Whole-Genome Sequencing (WGS)

Advancements in sequencing technology have significantly reduced the time and cost associated with WGS [90], (Table 2, Figure 3 and Figure 4). WGS of domestic animals is highly beneficial for identifying QTLs, candidate genes, MAS, and SS, and for understanding their relationships with reproductive and production traits, animal health, and welfare. This technology aids in numerous animal production practices and helps elucidate the genetic basis of diseases and GWAS applications [171].

More recently, several whole genomes have been investigated for many livestock species with big data about the history of these species and their domestication; this process also investigated most of the economic traits and GWAS in farm animals (Table 2).

#### 7.6.1. The Genome-Wide Studies (GWS)

A primary objective in animal breeding is to select individuals with high breeding values for key traits to become the parents of future generations. The success of GWS in identifying sequence variations linked to important complex traits has spurred interest in SNP genotyping for detecting QTLs, candidate genes, and for implementing GS [172].

The advent of SNP genotyping, paired with advanced statistical methods for predicting breeding values, has greatly enhanced the application of WGS and genomic studies in livestock. This has led to the extensive implementation of GS in various animal species by the scientific community [92,128]. For example, in Texel sheep, there is a 166-fold coverage, and a length of 2.61 Gb, and the annotation involves 20,645 protein-coding genes [173].

#### 7.6.2. Genome-Wide Association Studies (GWAS)

GWAS involve quickly scanning genetic markers across entire genomes of multiple individuals to identify genetic differences linked to specific traits. Once new associations are found, researchers can use this information to develop enhanced strategies for improving traits in animal populations [174]. It is worth mentioning that this approach was recently used to improve several economic traits in different animal species: buffaloes, beef cattle, dairy cattle, sheep, goats, rabbits, and pigs. Such investigations are particularly useful in finding genetic variabilities that contribute to quantitative traits [93,174].

In this regard, the large amounts of high-density SNP data generated by NGS, to implement WGS can also be utilized for GWAS to identify genomic regions or GM associated with target traits that are related to population-wide linkage disequilibrium (LD) [175]. One of the most relevant parameters to apply GWAS in breeding programs is LD. LD is known as a non-random association of alleles at two or more loci. It is influenced by the breeding system, the pattern of the geographic subdivision, and the population history [176]. The density of markers required for successful GWAS depends on the extent of LD through the genome. The small value of LD may require a high density of markers to capture most of the variation in the families. The persistence of LD has been estimated and evaluated in many domesticated species. It is necessary to keep on characterizing LD in many collections of populations. To obtain high convenient accuracies of prediction when utilizing many breeds, there is a requirement not only for high LD between QTL and markers in each strain/breed but also for a high consistency of gametic phase between them across breeds [42].

For GWAS, many alternative methods of statistical analysis have been utilized [177] besides using the SNP chips. However, most studies have utilized single SNP models where each SNP is tested separately, as a fixed effect in the animal model of BLUP, to account properly for population structure and to use the polygenic effect with the relationships of pedigree [178]. Moreover, SS related to a hot and dry climate and metabolic traits were identified using WGS data by XP-CLR analysis [89]. Also, the information from WGS was successfully utilized to discover the regions that are under different pressures of selection in terms of reproductive traits [179].

More recently, Chen et al. [180] conducted GWAS with the help of LD correlation to narrow down the findings to obtain corresponding candidate genes on fifteen body dimension (size) traits utilizing autosomal SNPs derived from the WGS of 131 Yunling and 31 Brahman cattle breeds and identified twenty significant loci, which implicated eighteen candidate genes (*BMP5*, *LCORL*, *CNTNAP5*, *EEPD1*, *CACNA1C*, *RAP1GDS1*, *PDLIM5*, *HTR2A*, *LIMCH1*, *GRM8*, *F3*, *HAVCR1*, *CCNH*, *MCFD2*, *C5H12orf66*, *SUDS3*, *CTNNA3*, and *CCDC6* genes). They reported that there were seven significantly associated loci with body size on BTA28, BTA17, BTA15, BTA12, BTA7, BTA5, and BTA4. Also, they found a significant SNP in the *LCORL* gene for ischium width and a significant SNP was located upstream of the *BMP-5* gene, which related to chest width. Their findings showed that the LIMCH1 locus was associated with forehead size, and the CNTNAP5 locus was associated with hip-cross height in Brahman cattle. The GWAS findings provide genetic insights into selection and variation and will quicken future endeavors aimed at livestock improvement [174].

### 7.7. Detection of SS

High-throughput SNP genotyping and NGS have greatly contributed to identifying SS in livestock populations [181]. SS, or the unique genetic patterns left by natural or artificial selection, result from changes, reductions, or eliminations of genetic variation near causative variants under selection pressure. These regions often contain important sequence variants crucial for genomic selection (GS) [134,182]. Identifying SS is essential as it helps reveal mutations and genes linked to phenotypic traits without direct measurement [182]. Advanced genomic tools and high-throughput SNPs have enhanced the exploration of SS and the genomic diversity arising from selective pressures and environmental adaptations [134]. The detection of SS aids in understanding and identifying mutations and genes associated with key traits in livestock species. Moreover, the detection of SS in the whole genome can help improve defining candidate genes and elucidate mechanisms for selection [183]. Thus, utilization of SS could help to design and update the programs of animal breeding. Moreover, assessing levels of polymorphism and genetic diversity within a population is essential as genetic variation serves as the foundation for implementing GS in animal breeding [184]. Additionally, identifying genomic regions related to and affected by normal selection is necessary to understand the history of artificial and natural selection in populations. Also, the domestication of these regions provides insights into some important biological pathways related to economic and qualitative traits along with their goals of breeding [185].

### 7.8. The Contribution of Advanced Molecular Tools to the Detect of SS in Livestock Populations

In small ruminants, Bayesian methods were used to obtain SS in regions that contain many genes associated with the immune system of the Valdostana mountain goat breed in Italy [186]. Zhao et al. [183] tested three sheep breeds (Sunite, German Mutton, and Dorper) utilizing the 50K Ovine SNP chip to detect positive SS, and discovered 42 regions. Those genomic regions harbor necessary genes such as *IGF-1*, *SST*, *BMP-3*, *TTN*, *DNAJC27*, *FAP*, *MYO1G*, *TSHR*, *MYOZ-3*, *MYO-10*, *SIRPA*, *BMPR2*, *MEX3C*, *POMC*, *PMCH*, *SLC44A-5*, *NCPAG*, *LEPREL-1*, *VGLL-3*, *PAX-7*, *GDF-1*, *KBTBD5*, *HOX*, and *SIRPA*, which are related to muscle and bone development, fat deposition, and body size. On the other hand, there are many genes related to reproduction traits, such as *PCB-1*, *LYZL-4*, *LRPPRC*, *LH-B*, *POU1F-1*, *EPT1*, *FSH-R*, *TDRD-1*, *SPATA-17*, *CIB-4*, and *FGFR-2*, to the developing of hair follicles, such as *EDAR*, and related to coat colors, such as *MITF* and *KITLG*. Many of those genes are found in the genome of humans, cattle, and chickens [42]. Also, Johnston et al. [187] reported that the relaxin-like receptor-2 (*RXFP-2*) gene in sheep was associated with the absence/presence of horns (an important feature in breed definition), and an SNP surrounding this gene appeared as a strong selection signal in an analysis involving seventy-four sheep breeds.

In cattle, Cheruiyot et al. [188] investigated the genotypes of 839 individual dairy cows at 150.000 SNP loci utilizing the Gene seek Genomic Profiler High-Density (GGP-HD) SNP array. They explored evidence of SS by utilizing three statistical methods (XP-EHH, iHS, and pcadapt). SS analysis identified about 108 candidate regions in the studied dairy cattle. Annotation of those regions showed interesting genes that were potentially under strong positive-selection involving *ABCC-2*, *ABCG-2*, *XKR-4*, *TGS-1*, *LYN*, *TOX*, *KIT*, *HERC-6*, *CHCHD-7*, *PLAG-1*, *LCORL*, and *NCAPG*, which are involved in several biological pathways underlying adaptation and production processes. On the other hand, several investigations reported that the BTA6 chromosome harbors at least three QTLs that affect milk production and composition in cattle [189]. As for beef cattle, many SS have been detected where mutations in the *GDF-8* gene were related to the double-muscled phenotype (Table S3). Also, Yurchenko et al. [190] studied nine Russian cattle breeds raised in harsh climates utilizing GGP-HD (150K), and they identified the candidate regions and SS to be under selection in the genomes of studied cattle. After comparing their findings to those for other breeds of Asian and European origin, they found novel candidate genes that could strongly be related to economic traits, environmental adaptations, and domestication in cattle. They found SS near major genes association with economic traits, such as growth (e.g., XKR-4), reproduction (e.g., CSF-2), and milk production (e.g., *DGAT-1* and *ABCG-2*) and other SS were associated with adaptations to extreme environments (e.g., AQP-5, *RETREG-1*, and *RAD-50*). Peripolli et al. [191] used WGS to detect SS in Brazilian cattle (Gir, Caracu Caldeano, Crioulo Lageano, and Pantaneiro breeds). They reported that SS retrieved from the de-correlated composite of the multiple-signals (DCMS) statistic method provided a comprehensive set of candidate genes and revealed QTLs disclosing cattle production, reproduction traits, and adaptation to harsh environments.

In horses, Gurgul et al. [192] reported that the availability of new molecular tools, especially high-throughput genotyping approaches, created the opportunity to investigate the genetic variation on the whole genome, thus detecting important genome regions and SS. They used the “Neogen-Equine SNP-array Chip” (Illumina), involving probes for 65,157 SNPs to detect SS between six different horse breeds raised in Poland: light horses (Arabians and Malopolski-horse), primitive horses (Hucul and Polish-Konik), and draft horses (Sokolski and Sztumski). The analysis of the most pronounced selection-signals found in their study allowed them to discover several genomic regions, SS, and candidate genes associated with processes necessary for breed phenotypic differentiation and related to heart functioning, motor coordination, fertility, energy homeostasis (during physical effort), and disease resistance, for example, the association of loci on ECA3 (spanning *NCAPG* and *LCORL* genes) with those traits as well as ECA11 (spanning *LASP1* gene) with the regulation of body size in the draft horses. Also, their results identified a robust SS in the blue dun-colored Polish Konik breed at the locus of *TBX-3* gene, which was recently discovered to be responsible for dun-coat color dilution.

Additionally, there are several SS related to meat production (Table S1), body size (Tables S2 and S3), milk production and composition (Table S5), fiber production (Table S6), and coat color and skin sensitivity (Table S7) in many species that have been detected and that are now utilized in animal breeding programs. Other tests of SS were performed on different species such as Alpine goats for milk production (Table S5), Chinese beef cattle, Wagyu Angus cattle, Thari goat, Blanca de goat, and Rasquera goats and pigs for meat production (Table S2), and for fiber production in cashmere and Nubian goat breeds (Table S6). It is worth mentioning that the high number of significant regions identified in different species could be attributed to the long-term selection practiced for meat and milk production in these species [142].

### 7.9. Detection of Selective Sweeps

Selective sweep alludes to a procedure by which a new advantageous mutation reduces or eliminates variation in linked-neutral sites as it increases in frequency in the population [193]. Nowadays, the advent of NGS technologies provides a new dimension to such genome scans and detect selective sweeps [194]. Differentiation in genomic regions surrounding useful mutations has led to current strong selection. Identification of mutations and regions under selective sweeps may reveal the important genes underlying phenotypic polymorphisms. Statistical methods pick up some patterns of polymorphism by the selection of an important and useful mutation [195].

In this regard, domestication and artificial selection led to the creation of unique breeds and biological types of several livestock populations, undoubtedly altering the patterning of variation within their genomes [196]. High-strong selection led to fixing advantageous large-effect mutations underlying breed characteristics, domestic ability, or productivity, creating selective sweeps in which variation was lost in the flanking-region of the chromosome for the selected allele. Selective sweeps have recently been specified in the genomes of humans and numerous livestock species including cattle, sheep, goats, chickens, horses, and dogs [196,197]. More recently, many studies have been performed on different animal species with important selective sweeps. Here, we mentioned the most important recent investigations with important detected selective sweeps in different animal species: (1) in small ruminants, Guo et al. [198] reported that one region of a selective sweep is probably related to body size traits in the Nanjiang Yellow goat breed that is located at chromosome 6, between 112.10 and 112.20 Mb, with high differentiation and low heterozygosity; this region involves the *LDB-2* gene, which plays like a master regulator of trans-endothelial migration of atherosclerosis and leukocytes. In the investigation by Álvarez et al. [197], genomic data for 184 West African dwarf/WAD (Djallonké) sheep were generated utilizing medium-density SNP Chips (MD-SNP-ship/Ovine 50 K SNP-Bead-Chip “Illumina”) with the help of three statistical methods (XP-EHH, iHS, and nSL) that were applied to identify candidate sweep regions spanning genes putatively related to the adaptation of the studied sheep breed to its original environment. Their findings revealed about 207 candidate selective-sweep regions. Functional annotation analyses and gene-annotation enrichment led to the identification of three different statistically significant functional clusters involving twelve candidate genes—(a) cluster 1 (*ALB*, *GC*, *AFP*, and *AFM* genes), (b) cluster 2 (*MC2R*, *CIB1*, *MC5R*, and *CAV1*), and (c) cluster 3 (*LDLRAD4*, *LRP11*, *CFI*, and *VLDLR*)—which associated with metabolic response to stress (metabolic stress, thermotolerance, and regulation of oxidative). It is worth mentioning that these detected regions in sheep were similar to bovine-chromosomal regions carrying many QTLs for cattle trypan tolerance.

(2) In cattle, Ramey et al. [196] utilized BovineSNP50 data to identify about 28 putative sweep regions across 6373 individuals from 14 different cattle breeds (Bos taurus). Also, Affymetrix-BOS-1 pre-screening assay analysis for 5 out of 14 breeds was used to identify about 85 regions and validate the five regions identified. They reported that there were several candidate genes located within those regions that probably had historically been under strong selection, involving the behavior, stature, coat color, horned/polled status, and ear shape. Also, Peripolli et al. [191] discovered selective sweeps in Brahman cattle related to body dimensions using composite nucleotide diversity, likelihood ratio, and integrated-haplotype score. In this regard, Mariadassou et al. [199] used WGS to detect selective sweeps in French Limousin cattle (10 WGS of bull calves). They identified 171 different candidate selective sweeps, and 57 regions out of 171 that contained 68 candidate genes (those worth mentioning were the *MSTN*, *RUNX-2*, and *NCKAP-5* genes), contributing to important phenotypes in the studied breed. Also, they determined the causative regulatory polymorphisms in *RUNX2* and *GRIK3* genes. Several QTLs were also detected within candidate selective sweeps, including QTL associated with carcass weight and shear-force.

(3) In pigs, recently, Zhang et al. [200] generated 28 sets of WGS data for Danish large-white pigs with a 25-fold depth using the HiSeq X10 system (Illumina), thus, resulting in about 12,000,000 high-quality SNPs for each pig. Subsequently, these data were combined with the WGS of 23 European and 27 Duroc wild boars to investigate the selective sweeps with the help of 2 complementary methods (*Fst* and *iHS*). They identified 67 candidate selective-sweep regions as the signatures of parallel-selection. The candidate genes in these regions were mainly related to growth rate, body size, and sensory perception. Thus, these findings may help researches better understand the selection and parallel-selection processes of different pig breeds.

These discoveries will contribute to improving the comprehension of the targets and mechanisms of artificial selection and will facilitate the interpretation of GWAS performed on different livestock populations [191,199].

All of the above created a new concept, which is genomic selection (GS). GS is a large-scale extension of MAS, utilizing a substantial number of genetic markers. During GS, the effects and roles of each marker are estimated simultaneously. Thus, this facilitates the designing of new effective animal breeding programs alongside developing novel markers-based models for genotype evaluation. GS has become a necessary approach in animal breeding. The molecular tools that can detect genetic differentiation at the DNA sequencing level and the morphological protein markers have removed the chromosomal limitations without affecting their unique properties. The genetic differentiation and polymorphism at the DNA level have provided a large number of MM that have potential utility for application in animal breeding using several approaches [42,165].

## 8. Genomic-Selection Applications

The application of wide-genetic-markers (WGM) in animal breeding was initially proposed by Meuwissen et al. [201]. Traditional MAS relied on a relatively small number of genetic markers identified through research findings and primarily controlled testing analyses. Those markers were recognized from research results, primarily analyses of controlled tests [202].

The extent of MM included in genomic evaluations varies based on the method used. GS posits that markers should account for all genetic differences in traits. However, polygenic effects might contribute to genetic variation that markers cannot explain. Ultimately, GS aims to use genotypes, characterized by polymorphisms, to select desired phenotypes [203].

GS can significantly enhance traits that traditional methods aim to improve. Certain crucial traits, such as carcass traits and disease resistance, are either costly or challenging to measure. Others, like milk composition, production, and carcass characteristics, are only measurable in one sex or later in an animal’s life. Modern techniques like GS can address these limitations in genetic enhancement [204], (Figure 5).

GS can enhance resilience in animal species by boosting production, facilitating gradual and proper adaptation, and promoting resistance to fatal diseases [204]. This encompasses breeding for resistance to diseases, parasites, fly-strike, and facial eczema [205]. Additionally, GS offers ethical advantages by reducing the incidence of susceptible and suffering individuals in future generations. Current research is focusing on enabling GS for crucial biological traits such as methane emissions and feed efficiency, which are costly to measure (with measuring not easy to implement on farms), making GS a potentially promising alternative [193].

GS leverages genotypic, phenotypic, and pedigree data, offering an opportunity for implementing breeding programs in farm animals to enhance meat (Table S1), fiber (Table S6), and milk production (Table S5). It also addresses traits that are challenging to manage with traditional methods, such as reproduction (Table S4), breeding seasonality, longevity, meat quality, and carcass composition (Tables S1 and S8). The viability of GS for small ruminants has been assessed in Australian mutton breeds [206], French dairy sheep [207], and French dairy goats [207]. GS methods have been successfully adopted in dairy cattle breeding programs, leading to a reduction in the generation interval. Despite sheep and goats having shorter generation intervals compared to cattle and buffaloes, further reduction is necessary. This decrease, along with increased selection intensity, boosts genetic gains per year, lowering costs and raising productivity [203].

In GS, SNPs are tested for their high-density effects using a model that simultaneously fits each SNP, considering these effects as random variables. Various Bayesian models have been refined to apply statistical estimation through the Monte Carlo Marko Chain (MCMC) methodology [208].

### The Methodology of Genomic Selection

To implement genomic selection (GS) in any animal population, certain requirements must be met: (1) a large sample size for each genotype, (2) availability of phenotype data specific to each genotype, and (3) appropriate statistical methods for accurate genetic prediction (Figure 5). Assuming the breeding program is optimal, it should aim for (a) accurate genetic evaluation systems for relevant phenotypes, (b) breeding objectives aligned with target traits, and (c) a breeding scheme that ensures long-term sustainable genetic gains [209].

**Figure 6:**
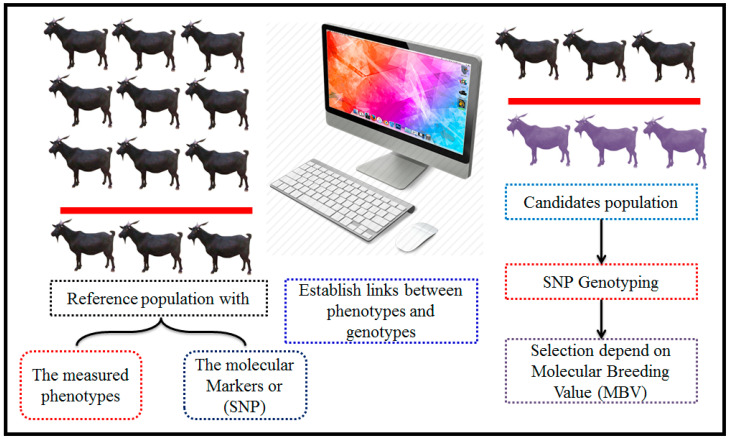
The methodology of genomic selection. This Figure outlines the step-by-step methodology of genomic selection in animal breeding programs. The process begins with comprehensive phenotypic and genotypic data collection from a well-characterized reference population. High-throughput SNP genotyping is conducted to gather detailed genomic data. The next step involves estimating the effects of individual SNPs on target traits using sophisticated statistical models. Finally, these selected individuals are used in breeding programs to enhance genetic gain and improve specific traits across the population, thereby optimizing the overall breeding strategy. This methodology represents a powerful approach to accelerating genetic improvement by efficiently leveraging genomic information.

In general, (1) the data collected will serve as a reference for developing new statistical models to estimate SNP effects on target traits, (2) the outcomes will be equations for predicting GEBV [126], (3) in the absence of accurate phenotypes, GBVs of new individuals can be estimated from these prediction equations based on SNP genotypes, and (4) the accuracy of GEBV will depend on the heritability of the traits and the population size [140], (Figure 6).

## 9. Future Perspectives

As the field of genomic selection and molecular marker technology continues to advance, the potential for enhancing livestock breeding programs is vast. Future developments are expected to focus on improving the precision and scalability of these technologies. This will involve integrating high-throughput sequencing data with advanced bioinformatics tools to provide more accurate genomic predictions. The use of machine learning and artificial intelligence in analyzing complex genetic data could offer new insights into trait heritabilities and interactions, thereby further optimizing selection strategies. Additionally, as we enhance our understanding of gene edits through CRISPR and other gene-editing technologies, there is potential for directly editing genetic material to enhance desirable traits, raising prospects of significantly accelerated breeding cycles.

Concurrently, expanding our reference population databases with diverse global data will also play a crucial role. There is a growing need to incorporate genomic information from underrepresented breeds, especially those adapted to unique environmental challenges. This could lead to improved resilience and sustainability in breeding programs, particularly in the face of climate change and shifting agricultural practices. Moreover, ethical considerations and regulatory frameworks will need to keep pace with these technological advancements to ensure responsible and equitable application. Investing in education and capacity-building efforts around these innovations is essential to ensure that all stakeholders, from smallholder farmers to large-scale producers, can benefit equally from these advancements in genomic selection and molecular tools.

## 10. Conclusions

The current investigation highlights the pivotal role of advanced molecular markers and genomic selection in modern livestock breeding. These molecular tools offer significant advantages, such as enhancing challenging traits, including those that are sex-limited, possess low heritability, or are measured later in an animal’s life. They can be implemented early in life and improve the accuracy and intensity of selection, thereby boosting genetic gain by reducing the generation interval. Molecular markers also allow for the clarification of genetic differences at the DNA level, supporting their extensibility to traits recorded in reference populations, and are effective across diverse populations.

Our investigation provides foundational insights into the evolution and impact of molecular tools in livestock populations. It details animal genetic resource biodiversity and advanced molecular strategies, like SNP genotyping, GM, mt-DNA, MAS, candidate genes, QTLs, selective sweeps, WGS, and GWAS, culminating in genomic selection. This comprehensive knowledge is crucial for understanding and leveraging these technologies in animal production and genetics applications. Therefore, we consider this study a significant resource for researchers focusing on animal genetics and breeding.

## Figures and Tables

**Table 1:** Types of markers (molecular markers, biochemical markers, and morphological markers).

Type of Marker	Description	Techniques Used	Applications	Advantages	Limitations
Molecular Markers	DNA-based markers used to identify genetic differences between individuals.	- PCR	Gene mapping	High specificity	Can be expensive
		- RFLP	Genetic diversity studies	Detects minute genetic variations	Requires technical expertise
		- AFLP	Marker-assisted selection		
		- SSR	Parentage analysis		
		- SNP	Evolutionary studies		
		- RAPD			
Biochemical Markers	Proteins or enzymes linked to biochemical pathways, reflecting phenotypic traits.	Isozyme analysis	Assessment of functional variations	Less expensive than DNA markers	Limited resolution
		Protein electrophoresis	Genetic mapping of certain traits	Direct link to gene expression	Environmentally influenced
			Identifying metabolic pathways		
Morphological Markers	Observable phenotypic traits such as seed shape, flower colour, or plant height.	Traditional breeding methods	Early genetic studies	Easy and inexpensive	Influenced by environment
		Visual identification	Plant breeding	No need for complex technology	Often controlled by multiple genes
			Taxonomy Conservation programs		

**Table 2:** The early sequenced genomes for some animal species.

Species	Genome Size (Gb/Mb)	Year	Reference
Fruit Fly (*Drosophila melanogaster*)	0.18	2000	[54]
Mouse (*Mus musculus*)	2.6	2002	[55]
Dog (*Canis familiaris*)	2.4	2003	[56]
Chicken (*Gallus gallus*)	1.05	2004	[57]
Norway Rat (*Rattus norvegicus*)	2.75	2004	[58]
Sea Urchin (*Strongylocentrotus purpuratus*)	0.81	2006	[59]
Elephant Shark (*C. milii*)	910-Mb	2007	[60]
Monkeys (*M. mulatta*)	3.09	2007	[61]
Cat (*Felis silvestris catus*)	2.7	2007	[62]
Platypus (*Ornithorhynchus anatinus*)	1.9	2008	[63]
Pig (*Sus scrofa*)	2.2	2008	[64]
Sheep (*Ovis aries*)	2.78	2009	[65]
Cattle (*Bos taurus*)	2.91	2009	[66]
Horse (*Equus caballus*)	2.47	2009	[67]
Amphibians (*Xenopus tropicalis*)	1.7	2010	[68]
Giant Panda (*Ailuropoda melanoleuca*)	2.4	2010	[69]
Zebra Finch (*Taeniopygia guttata*)	1.2	2010	[70]
Turkey (*Meleagris gallopavo*)	1.1	2010	[71]
Camel (*Camelus dromedarius*)	2.2	2011	[72]
Tammar Wallaby (*Macropus eugenii*)	3.1	2011	[73]
Green Anole Lizard (*Anolis carolinensis*)	1.78	2011	[74]
Atlantic Cod (*Gadus morhua*)	0.83	2011	[75]
Naked Mole Rat (*Heterocephalus glaber*)	2.7	2011	[76]
Orangutan (*Pongo abelii*)	3.08	2011	[77]
Goat (*Capra hircus*)	2.66	2012	[78]
Bactrian Camel (*Camelus bactrianus*)	2.38	2012	[79]
Olive Baboon (*Papio anubis*)	3.1	2012	[80]
Wild Duck (*Anas platyrhynchos*)	1.07	2013	[81]
Coelacanth (*Latimeria chalumnae*)	2.91	2013	[82]
Sea Lamprey (*Petromyzon marinus*)	0.81	2013	[83]
King Cobra (*Ophiophagus hannah*)	1.44	2013	[84]
Dung Beetle (*Onthophagus taurus*)	0.38	2022	[85]
Cherry Salmon (*Oncorhynchus masou*)	2.4	2024	[86]

**Table 3:** Types of molecular marker (genetic markers) tools.

Type	Method		Most Applications	Ref.
Biochemical markers	Isozymes	Allozymes	Animals	[97]
First Generation Markers	RAPD	Random Amplified Polymorphic DNA	Cattle and Sheep	[98]
	SCAR	Sequence Characterized Amplified Regions	Chickens	[99]
	STS	Sequence Tagged Sites	Plants	[100]
	AP-PCR	Arbitrary Primed PCR	Cattle	[101]
	DAF	DNA Amplification Fingerprinting	Cattle and buffalo	[102]
	RFLP	Restriction Fragment Length Polymorphism	Sheep and goats	[88,103]
Second Generation Markers	AFLP	Amplified Fragment Length Polymorphism	Humans and animals	[104]
	SSLP	Simple Sequence Length Polymorphism	Fish	[105]
	ISSR	Inter Simple Sequence Repeat	Sheep	[106]
	VNTRs	Variable Number of Tandem Repeats	Cattle	[107]
	RAMPO	Random Amplified Micro satellite Polymorphism	Humans and animals	[108]
	CAPs	Cleaved Amplified Polymorphic Products		
	ASAP	Allele-Specific Associated Primers	Mouse	[109]
Third Generation Markers	EST	Expressed Sequence Tag markers	Pigs	[110]
	SNP	Single Nucleotide Polymorphism	Goats	[111]
	MITE	Miniature Inverted Repeat Transposable Elements	Plants	[112]

## Data Availability

All data generated or analyzed during this study are included in this manuscript and its information files.

## Supplementary Materials

The following supporting information can be downloaded at: https://www.mdpi.com/article/10.3390/vetsci11120627/s1, Supplementary information File S1: The list of databases used in the current investigation, Supplementary information File S2: Supplementary Tables.
