# Supplementary material for: The Evolution and Role of Molecular Tools in Measuring Diversity and Genomic Selection in Livestock Populations (Traditional and Up-to-Date Insights): A Comprehensive Exploration
Source: Vet Sci. 2024 Dec 6;11(12):627. doi: 10.3390/vetsci11120627 (PMC11680231; doi:10.3390/vetsci11120627)
Supplement: Supplementary file 1 [file vetsci-11-00627-s001.zip › vetsci-3256180-supplementary/Supplementary information files/Supplementary information file (1); The list of databases used in the current investigation.pdf]

Supplementary information file (1); The list of databases used in the current investigation

The list of databases used in the current investigation includes:

1. FAO-Database (https://www.fao.org).
2. Animal QTL-Database: (https://www.animalgenome.org/cgi-bin/QTLdb/index).
3. National Animal Genome Research Program (NAGRP): (https://www.animalgenome.org).
4. FAANG (Functional Annotation of Animal Genomes): (https://www.faang.org).
5. Cattle-QTLdb: (https://www.animalgenome.org/cgi-bin/QTLdb/BT/index).
6. Sheep-QTLdb: (https://www.animalgenome.org/cgi-bin/QTLdb/OA/index).
7. Goat genome browser: (https://www.ensembl.org/Capra_hircus/Info/Index).
8. International-Goat Genome-Consortium: (https://www.goatgenome.org).
9. Var. Goats project: (https://gsejournal.biomedcentral.com/articles/10.1186/ s12711-021-00659-6).
10. ChickenQTLdb: (https://www.animalgenome.org/cgi-bin/QTLdb/GG/index).
11. Pig-QTLdb: (https://www.animalgenome.org/cgi-bin/QTLdb/SS/index).
12. Horse-QTLdb: (https://www.animalgenome.org/cgi-bin/QTLdb/EC/index).
13. BioGPS Livestock Genomics: (http://biogps.org/#goto=genereport&id=70).
14. International Bull Evaluation Service: (https://interbull.org/ib/interbullcentre).
15. Animal Genome Size Database: (http://www.genomesize.com).

Supplementary information file (1); The list of databases used in the current investigation

16. Genome-Informatics-Resources: (https://www.animalgenome.org/bioinfo). Adapt-Map project.
