# Supplementary material for: The Evolution and Role of Molecular Tools in Measuring Diversity and Genomic Selection in Livestock Populations (Traditional and Up-to-Date Insights): A Comprehensive Exploration
Source: Vet Sci. 2024 Dec 6;11(12):627. doi: 10.3390/vetsci11120627 (PMC11680231; doi:10.3390/vetsci11120627)
Supplement: Supplementary file 1 [file vetsci-11-00627-s001.zip › vetsci-3256180-supplementary/Supplementary information files/Supplementary information file (2); Supplementary Tables.pdf]

Supplementary information file (2); Supplementary Tables

Table S1. Candidate genes associated with meat production, quality and their main biological functions.

Gene name	Gene symbol	Biological process	The related traits	*Ref.*
**Growth Hormone**	*GH* (19q22)	Produces GH hormone, regulates the birth and weaning weights, and manages postnatal growth.	Growth, metabolism, postnatal growth, milk yield in vertebrates.	[1] [2] [3]
**Arylalkylamine N-Acetyltransferase**	*AANAT*	Encodes the enzyme of penultimate in the production of melatonin, has an effect on reduced lipid-peroxidation, and protects the (long-chain PUFAs)	Fatty aldehyde, total fatty acids collagen muscle content, omega-3 PUFA, pH at ten days postmortem, flavour	[4-6]
**Insulin-Like Growth Factor-I**	*IGF-I* (5q31)	Produces IGF-I hormone through the effect on the neuroendocrine and their regulate pathways, thus, the metabolism and postnatal growth in mammals.	Metabolism, growth, reproduction	[7] [8,9]
**Calpain - 1**	*CAPN-1*	- Associated with the protein breakdown in meat Post-mortem,<br>- Regulate adipocyte differentiation,<br>- Related to high free fatty acid levels	Meat tenderness	[10-12] [10,13]
**Leptin**	*LEP* (3q33)	Provides instructions to produce the leptin hormone	Body temperature regulation, feed conversion efficiency, energy expenditure, growth development, metabolic balance in the whole body	[14,15]
**Calpastatin**	*CAST*	Inhibitor of calpains	Juiciness, tenderness, water-holding capacity, fatty acid profile in the muscles, the colour of the meat	[4,16] [17,18] [19]

Supplementary information file (2); Supplementary Tables

Table S1 Continued.

**Caprine Pituitary-specific Transcription Factor-1**	*POU1F1* (*PIT-1* or *GHF-1*) (1q21-q22)	Regulate prolactin, TSH and GH hormones	Affect carcass characteristics, Growth, the effect on fibre and milk	[20] [21] [20]
**Caveolin 3**	*CAV-3*	Provides instructions to produce caveolin-3 protein, in the membrane surrounding muscle cells. This protein is the main important component of caveolae, (small pouches in the membrane of muscle cells)	Necessary role in the development of the T-tubule system in the muscle	[22] [23] [24]
**CGG triplet repeat-binding protein 1**	*CGGBP-1*	Progression and regulation of the cell cycle	Many carcass traits	[25] [26] [27]
**Corticotrophin-releasing a hormone**	*CRH*	The indirect release of glucocorticoid growth inhibitors	Growth, Carcass yield	[28] [29]
**Myostatin**	*MSTN*	Nine (bp) variations in this gene and their new amino acids, variation gave rise to double muscling in cattle, point mutations of this gene influenced the improvement of average daily gain in chickens and pigs.	A candidate gene for muscle growth and development in most domestic animals	[30] [31] [32] [33]
**Kappa-casein**	*CSN-3*	Encodes a lactoprotein	Carcass traits, milk traits; fat and protein composition, cheese performance, and coagulation properties	[34] [35]
**Heat shock 27 kDa Protein-1**	*HSPB-1*	Involved in actin organization and stress resistance	Meat tenderness	[36] [37]

Supplementary information file (2); Supplementary Tables

Table S1 Continued.

**Pro-opiomelanocortin**	*POMC*	Increase synthesis of the αMSH hormone, which reduces appetite by binding to the melanocortin-4 receptor (MC4-R)	Growth, carcass yield	[29] [38]
**Bone Morphogenetic Protein**	*BMP*	Bone formation when implanted within the soft tissue (in vivo)	Cell differentiation, Embryonic development, apoptosis, homeostasis, and repairing of various tissue patterning, the female fertility	[39] [40] [41]
**RAR-related orphan receptor alpha**	*RORA*	Regulation and promotion of lipid homeostasis of myogenesis in the skeletal muscles.	The disruption in this gene has been related to severe obesity in humans	[42] [43]
**Thyroglobulin**	*TG*	Glycoprotein hormone precursor of the Triiodothyronine (T3) and Thyroxine (T4), plays a necessary role in the regulating of lipid metabolism.	The marbling	[44] [45] [46]

Supplementary information file (2); Supplementary Tables

Table S2. Candidate genes and signatures of selection associated with meat production for genomic selection.

	Genes/Sites	Sites/chromosomes (CHR)	Traits	Species/breeds	*Ref.*
1		25 regions on (8 chromosomes)			
2	Regions	121 regions and/or SNPs on (27 chromosomes)	TSHB is associated with muscular functionality.		[47]
3		24 regions on (16 chromosomes)	AMPD1 is involved in the deamination of AMP in skeletal muscles and is associated with many traits such as; body weight and heart girth.		
4	*TSHB* (Thyroid-stimulating hormone beta) *NRAS* (Neuroblastoma RAS viral onco-gene homolog) *AMPD1* gene (Adenosine monophosphate-deaminase-1),	Two regions on CHR-3, and 18 were detected and contained 18 genes including TSHB, NRAS, and AMPD1		Meat goat breeds Chinese beef cattle	[48]
5	*EFEMP-1* (EGF Containing Fibulin Extracellular Matrix Protein -1)	CHR- 11	Associated with conjugated linoleic lipid contents in the meat	Wagyu Angus cattle	[49]
6	*CAPN10* (Calpain-10)		Play important roles in muscle development and growth, differentiation, and myoblast fusion,	-Thari, Blanca de, Rasquera - Pigs	[50]
7	*RNPEPL1* (Aminopeptidase-1)		associated with meat tenderness		[51]
	LYPLA1, *XKR4* and *TMEM68* genes	BTA14	Associated with feed intake, growth phenotypes, marbling and fatness traits	Beef cattle	[52]

Supplementary information file (2); Supplementary Tables

Table S3. Candidate genes and signatures of selection associated with body size for genomic selection.

	Genes	Traits and Chromosomes (CHR)	Effects	Species/breeds	*Ref.*
1	*LCORL*	Body size<br>(OAR6)	Associated with SS linked to cattle body size	-Nubian goat (Egypt)<br>- Cattle and pigs (Europe)	[53]
2	*TGS1, LYN, PLAG1*	Stature	Associated with stature in human and cattle	Human and cattle	[54]
3	**A-** *CHCHD7, SMAD2,PLAG1*	Body size	Associated with body size in human, cattle, horse, and dog	Limousin cattle	[55]<br>[56]<br>[57]
	**B-***GDF5*			Angus and Simmental cattle breeds	
	**C-** *CDK6JAZF1, PRKG2*			Simmental,Belgian Blue	
	**D-** *GDF-8*	Double-muscled phenotype		Beef cattle	[58]
4	*PDGFRA, DGAT-1, MSTN,ZNF-521, GHR,CAPN-3, ABCG-2, IGF-I GAS-1, TMEM-130*	Body size<br>Body weight<br>Feed intake	Associated with many traits such as; body weight, body size, and feed intake		[56]<br>[59]
5	*LDB2*<br>(LIM Domain-binding Factor-2)	CHR-6 between<br>(112.10–112.20 Mb)	The master regulator of transendothelial migration of atherosclerosis and leukocytes	Nanjiang Yellow goat<br>(Chinese meat breed)	[60]
6	*BMP-2*	CHR- OAR18	Related to body size and skeletal morphology		[61]
7	*HMGA2*	CHR- OAR5			
8	*NPR2*	CHR- OAR1			
9	*NCAPG, LAP3*	CHR-OAR6	Associated with the height in cattle and humans		[62]
10	*SMAD1, BMPR1B, AKT1, TSC1*	A sgnificant relationship exists between the expression of RNA for these genes and genetic variability especially in (CpG) sites of methylation associated with body size variability in goat and sheep.			[63]

Supplementary information file (2); Supplementary Tables

Table S4. The activity of genes associated with reproduction.

Genes	Activity	*Ref.*
PAIP-2B, CCDC-64, EPB41L-5	Neurohypophyseal hormone activity	[64]
BIRC-6, TAOK-1, C6H-4 or f22, SGOL-1, SLC33A-1,SLC33A-1, C6H-4 or f22, TAOK-1	Photoreceptor activity, blue light photoreceptor activity	[65] [66] [67]
PAIP2, CLEC16A	Sex determination, mating type	[64]
PARD3B,FAT1, KDM4C	Spermatid development	[68]
FAT1, KDM4C, TPPP3, SBF1, PARD3B	Spermatogenesis; male gamete generation	[69]
KHDRBS-2	number of teats, pregnancy status	[64]
FOXL2 gene (Forkhead Box L2)	A transcription factor (TF) was special for proper reproductive activity in females. Also. Important for the establishment of the body axis, the developing of tissue from the three germ layers, metabolic processes, and cell cycle control. The mutation in this gene leads to the polled syndrome (PIS) syndrome thus, the absence of horns in both sexes in goat breeds. In addition, this mutation also leads to (XX) female-to-male sex reversal in a recessive manner.	[70] [71] [72]
MTNR1A gene (Melatonin Receptor 1A)	Mediates reproductive and fertility functions in the mammalian species. There was a polymorphism in (Exon 2) in ewes, this polymorphic site is related to reproductive seasonality in sheep, unlike goats.	[73] [74] [75]
SRY (Sex Determination Region of –Y-Chromosome)	A master candidate gene is responsible for the development of initiating genital, leading to testis formation. The mutations of this gene may lead to male-to-female sex reversal.	[75] [76]

Supplementary information file (2); Supplementary Tables

Table S4 Continued.

AMEL gene (Amelogenin)	Exists on the (X) chromosome (AMELX) and also exists on the (Y) chromosome (AMELY). It encodes a necessary protein in developing teeth in mammals. It is related to sex determination in humans, goats, deer, sheep and cattle.	[77] [78] [79] [80]
BMPR1B	Associated with goat prolificacy and litter size	[81] [82]
GDF9		[83]
EDA2R	Related to embryo dysplasia	[84]
HMGA2	Lack of this gene can cause abnormal fetal resource allocation, dwarfism, and cryptorchidism	[85]
MAGI1	Related to egg production performance in geese	[86]
NR6A1	NR6A1 has a necessary effect on oocytes, also, is critical for embryonic development, additionally, it is a transcriptional repressor by specifically binding to DR0 response elements.	[87-90]
AR	AR is related to the development of ovaries, also, is association with the vitality of sperm stores (in the testes).	[91,92]
PPP3CA, PLCB1	Many pathways were found related to the oocyte meiosis, estrogen signalling pathway, GnRH signalling pathway, and (Wnt) signalling pathway for these genes.	[93-96]
STK3, IGF2BP2, NPTX1, ANKRD17, CLRB, DPYD, PPP3CA, STK3, PLCB1	There are about 96 candidate genes were identified, which association with litter size in Dazu black goats including these genes.	[96] [97]

Supplementary information file (2); Supplementary Tables

Table S5. Candidate genes and signatures of selection associated with milk production and composition.

	Genes	**Sites**	**Traits**	**Species/breeds**	***Ref.***
1		11 regions on eight chromosomes			
2		286 regions and/or SNPs in all autosomes			
3		24 regions on 15 chromosomes			[98] [99]
4	*EFEMP1* (EGF containing fibulin like extracellular matrix protein 1)	4 regions on CHR-11, 13 and 14 included 20 genes that were detected among the *EFEMP1* gene	Milk production	Several dairy goat breeds	
**5**	*POU1F1*	CHR- 1q21-q22			[100]
**6**	*LEP*	CHR- 3q33			[101]
7	Casein (Casins cluster)	CHR- 6 between (85.95 and 86.25-Mb)			
*****	*αCSN1S1* (Alpha S1 casein)	CHR- 6 between (75-120 Mb), that contains the cluster of casein genes			[102] [103]
*	*CSN1S1*		The efficiency of protein synthesis.		[104]
*	*CSN1S2*		A high percentage of protein, and cheese yield.		[105]
*	*CSN2*		The sensitivity of calcium. Level of β–casein expression in milk.		[106]

Table S5 Continued.

*	*CSN3*		Associated with casein content and protein.		[104]
8	*ABCG-2*	CHR- 6	Milk composition and milk yield.	Murciano, Granadina, Argentata, dell'Etna Cattle & Sheep	[107]
9		CHR-1 between (110 and 130Mb)			
10	*LYPLA1*	The RGS20 and SOX17 genes		Dairy and Brahman cattle	[52]
11	*RGS20*	play roles in pubertal	Feed intake and weight gain		[108]
12	*SOX17*	development			
13	*SLC27A1*	CHR-7; 0.037 Mb	Fat content		
14	*DGAT1*	CHR-14; 0.111 Mb			
15	*PAEP*	CHR- 11; 0.034Mb	Protein content in	Saanen and Alpine breeds	[109]
*	*PLD2, ALOX12, GGT6, ALOX 15, and ALOX 12B genes*	CHR-19; 0.258 Mb	Protein yield		

Supplementary information file (2); Supplementary Tables

Table S6. Candidate genes and signatures of selection associated with fibre production for genomic selection.

	Genes/sites	Chromosomes (CHR)	Traits	Species/breeds	*Ref.*
1	18 regions on CHR- 11		Fibre production	Angora, Ankara, Cashmere	[110]
2	88 regions or/and SNPs on CHR-27				
3	24 regions on CHR-11				
4	34 genes	6 regions on CHR-6, 18, 25	Fibre production		
A	CHR- 3, 2, 5, 8, 10, 22 (47-48 Mb)		Fibre production	Cashmere	[111]
B	24 genes, including;<br><br>*-CUX-1*<br>(cut like homeobox1).<br><br>*-PLOD-3* (Procollagen-lysine, 2-oxoglutarate5-dioxygenase3)	CHR- 25 between (34.69-36.43 Mb)	1.CUX1 is associated with wavy hairs and curly whiskers in mice.<br><br>2. PLOD3 play a role in the formation of hair or their texture	Angora, Ankara	[111]<br>[112]

Supplementary information file (2); Supplementary Tables

Table S7. Candidate genes and signatures of selection associated with coat colour and skin sensitivity.

	Genes /chromosome (CHR)	Sites	Traits	Species/breeds	*Ref.*
1	*ADAMTS20*, *TIMP3* (TIMP metallopeptidase inhibitor 3)	CHR- 5 (70.0–70.5 Mb)	Required for melanoblast survival and mediating Kit signalling in skin colonization such as the belted white locus.	Angora, Ankara, Kil, Kilis	[113]
	SOX18 (SRY-box18), *ASIP*	CHR- 13 (53.0–53.5 Mb)	Plays a necessary role in hair follicles and blood vessel development during embryogenesis in dishevelled mice, a semi-dominant mutation characterized by coat sparseness.		[114]
	MC1R (Melanocortin 1 receptor)	CHR- 18 (15.50–16.25 Mb)	Controls the switch from eumelanin (Black and Brown) to pheomelanin (Yellow to Red).		[115]
2	CHR- 5<br>CHR- 9<br>CHR-13	(36.25–36.75 Mb)<br>(11.5–12-Mb)<br>(53–53.5 Mb and 62.75–63.25-Mb)	Coat colour and skin sensitivity.	Angora<br>Ankara	[110,116]
3	CHR- 8<br>CHR-22<br>CHR-29	(27.0–27.5 Mb)<br>(2.25–3.0 Mb)<br>(39.25–39.75 Mb)			
4	KIT		White coat in cattle.	Holstein cattle	
5	*DUSP22* (Dual specifcity phosphatase 22)<br>*IRF4* gene (Interferon Regulatory Factor-4)<br>*EXOC2* (Exocyst Complex Component -2)	CHR- 23 at the starting region between (0.025–0.35 Mb)	(1)- Hair colour/ skin sensitivity to the sun (the mutation in the intron number 4 of the IRF4 gene leads to enhance melanin in humans, while the lack of IRF4 gene in mice induced lighter coat colour).<br>(2)- These genes showed an association with coat colour.	*Human<br>*Mice<br>*Meigu goat	[120-117]

Supplementary information file (2); Supplementary Tables

Table S8. Identification of quantitative trait loci (QTL) for economically important traits in different livestock species.

Type of trait	Phenotype	Chromosomes	Specie /Breed	*Ref.*
	Fat depth	16	Texel sheep	[121]
	Muscle depth	11		
	Hot carcass weight (kg)	6, 9		[122]
		10, 18, 29	Cattle	[123]
	Leg weight (g)	6	Texel sheep	[121]
	Shoulder weight (g)	6, 9, 19		
**Carcass characteristics**	Rack weight (g)	6		
	Rib-eye area (cm^2)	6, 9		
	Back-fat thickness (mm)	4, 5, 9		
	Measurement of carcass fatness grade (mm)	4, 6, 13, 16		
	Lean-bone-rib (Kg) [1]	2	Beef and dairy cattle	[124]
	Lean-bone-leg (Kg)	5		
	Bone-prop-rib (Kg)[2]	5		
	Lean-prop-leg (Kg)[3]	10		
	Fifty-six carcass composition traits; including meat colour, carcass weight, muscling, fatness, tenderness, and fatty acid composition.	2, 3, 5, 6, 8, 11, 12, 14, 16, 18, 19, 20, 21, 22, 26	Merino, Poll Dorset, Border Leicester, Suffolk, white Suffolk, Texel, Corrie-dale and Coop-worth sheep breeds.	[125]
	Marbling (MAR)	3, 10, 14, 27	Cattle	[123]
**Carcass composition**	longissimus area (LMA)			
	Yield grade (YG)	2, 11, 14, 19		
	Estimated kidney, heart and pelvic fat (KPH)	15		
	Fat depth (FAT)	2, 7, 14		
	Meat tenderness	20, 29		

Table S8 Continued.

	Retail product yield (RPYD)	12, 18, 19, 29		
**Bone characteristics**	Bone area at the ischium	6	Scottish Blackface sheep	[126]
	Bone weight			
**Body conformation**	Head length	1,4	Angora goat	[127]
	Body length	8		
	Chest depth	2		
	Chest circumference	9		
	Width dimension	8, 12, 20, 25	Frizarta sheep	[128]
	Height dimension	9, 10, 11, 23		
	Length dimension	3, 19		
	Body length	2	Chinese Bulls	[129]
	Chest girth	2, 19		
	Foot angle, Teat placement, Udder depth,	6	Cattle	[130]
	Temperament	29		
	The general quality of feet and legs and udder	6		
	teat length	13		
	The general quality of feet and legs	23		
**Growth traits**	Birth weight	1, 16, 19, 22	Sheep	
		1, 3	Cattle	[123]
		4, 8, 17, 27	Angora goat	[131]
	Weaning weight	6, 21	Scottish Blackface sheep	[132]
		16, 19	Angora goat	[131]
		29	Cattle	[123]
	post-weaning gain and weaning weight	2, 3, 5, 8, 9, 17, 20	Zel and Lori-Bakhtiari sheep breeds	[133] [134]
	Body weight, average daily gain	1, 2, 5	Rayini goat	[135]

Table 8 Continued.

	Birth weight	14	Cattle	[136]
	Weight at 60 days	14		
**Fibre traits**	Quantity and quality	1, 2, 4, 5, 8, 13, 18, 20, 24	Angora goat	[137] [138]
		2, 5, 13	Rayini goat	[135]
**Resistance to diseases**	There are 18 locations from the 30 cattle chromosomes that appeared to contribute to disease resistance			
	Resistance to gastrointestinal nematodes	1, 3, 5, 7, 8, 9, 14, 16, 21, 22, 23, 26	Creole goat	[139]
		23	Angora, Cashmere goat breeds	[140]
	Resistance to trypanosomosis	17	Cattle	[141]
	Mastitis	6	Cattle	[130]
**Milk traits**	Milk yield	6, 14	Goat	[142]
	(MY)	6	Dairy Cattle	[143]
	Milking speed	29	Cattle	[130]
	Fat percentage	14, 20	Goat	[142]
	(F%)	6, 20	Dairy Cattle	[143]
	Protein percentage	3, 20	Goat	[142]
	(P%)	3, 6, 20	Dairy Cattle	[143]

[1]Lean-bone-rib = ratio of lean weight to bone weight (sum of vertebral column weight and other bone in forerib weight) in the forerib joint; [2]Bone-prop-rib = sum of weight of vertebral column and weight of other bone in the forerib joint as a proportion of forerib weight after full tissue dissection; [3]Lean-prop-leg = lean weight in the leg joint as a proportion of the leg weight after full tissue.

Supplementary information file (2); Supplementary Tables

References

1. Li, M.; Min, L.; Sun, G.; Pan, Q.; Shen, W.; Wang, G. Polymorphism analysis of the goat growth hormone gene in the 5'regulatory sequence. *Yi chuan= Hereditas* **2004**, *26*, 831-835.
2. Reinecke, R.; Barnes, M.; Akers, R.; Pearson, R. Effect of selection for milk yield on lactation performance and plasma growth hormone, insulin and IGF-1 in first lactation Holstein cows. *J. Dairy Sci* **1993**, *76*, 286-292.
3. Missohou, A.; Talaki, E.; Laminou, I.M. Diversity and genetic relationships among seven West African goat breeds. *Asian-australasian journal of animal sciences* **2006**, *19*, 1245-1251.
4. Dunner, S.; Sevane, N.; García, D.; Cortés, O.; Valentini, A.; Williams, J.; Mangin, B.; Cañón, J.; Levéziel, H.; Consortium, G. Association of genes involved in carcass and meat quality traits in 15 European bovine breeds. *Livestock Science* **2013**, *154*, 34-44.
5. Fagali, N.; Catalá, A. The effect of melatonin and structural analogues on the lipid peroxidation of triglycerides enriched in ω-3 polyunsaturated fatty acids. *Life sciences* **2007**, *81*, 299-305.
6. Perez, R.; Cañón, J.; Dunner, S. Genes associated with long-chain omega-3 fatty acids in bovine skeletal muscle. *Journal of applied genetics* **2010**, *51*, 479-487.
7. Zhang, C.; Zhang, W.; Luo, H.; Yue, W.; Gao, M.; Jia, Z. A new single nucleotide polymorphism in the IGF-I gene and its association with growth traits in the Nanjiang Huang goat. *Asian-Australasian Journal of Animal Sciences* **2008**, *21*, 1073-1079.
8. Shapiro, L.J.; Shapiro, D.B. Low Anabolic Profile in Assessing a Patient's Overall Hair Loss. In *Practical Aspects of Hair Transplantation in Asians*; Springer: 2018; pp. 687-698.
9. Bale, L.K.; Conover, C.A. Regulation of insulin-like growth factor binding protein-3 messenger ribonucleic acid expression by insulin-like growth factor I. *Endocrinology* **1992**, *131*, 608-614.
10. White, S.; Casas, E.; Wheeler, T.; Shackelford, S.; Koohmaraie, M.; Riley, D.; Chase Jr, C.; Johnson, D.; Keele, J.; Smith, T. A new single nucleotide polymorphism in CAPN1 extends the current tenderness marker test to include cattle of Bos indicus, Bos taurus, and crossbred descent. *Journal of animal science* **2005**, *83*, 2001-2008.
11. Patel, Y.M.; Lane, M.D. Role of calpain in adipocyte differentiation. *Proceedings of the National Academy of Sciences* **1999**, *96*, 1279-1284.
12. Orho-Melander, M.; Klannemark, M.; Svensson, M.K.; Ridderstråle, M.; Lindgren, C.M.; Groop, L. Variants in the calpain-10 gene predispose to insulin resistance and elevated free fatty acid levels. *Diabetes* **2002**, *51*, 2658-2664.
13. Page, B.; Casas, E.; Heaton, M.; Cullen, N.; Hyndman, D.; Morris, C.; Crawford, A.; Wheeler, T.; Koohmaraie, M.; Keele, J. Evaluation of single-nucleotide polymorphisms in CAPN1 for association with meat tenderness in cattle. *Journal of animal science* **2002**, *80*, 3077-3085.
14. Housekencht, C.; Baile, R.; Matteri, L.; Spurlock, M. The biology of leptin: a review. *J. Anim. Sci* **1998**, *76*, 1405-1425.
15. Whitley, N.C.; Walker, E.; Harley, S.; Keisler, D.; Jackson, D. Correlation between blood and milk serum leptin in goats and growth of their offspring. *Journal of animal science* **2005**, *83*, 1854-1859.
16. Barendse, W. DNA markers for meat tenderness. Patent PCT filed 8 February 2002. *US Patent Application 20040115678* **2002**.
17. Casas, E.; White, S.; Wheeler, T.; Shackelford, S.; Koohmaraie, M.; Riley, D.; Chase Jr, C.; Johnson, D.; Smith, T. Effects of calpastatin and μ-calpain markers in beef cattle on tenderness traits. *Journal of Animal Science* **2006**, *84*, 520-525.
18. Reardon, W.; Mullen, A.; Sweeney, T.; Hamill, R. Association of polymorphisms in candidate genes with colour, water-holding capacity, and composition traits in bovine M. longissimus and M. semimembranosus. *Meat science* **2010**, *86*, 270-275.

19. Riaz, M.N.; Ghaffar, A.; Khan, M.F.U. Calpastatin (CAST) gene polymorphism and its association with average daily weight gain in Balkhi and Kajli sheep and Beetal goat breeds. *Pakistan Journal of Zoology* **2012**, *44*.
20. Lan, X.; Pan, C.; Chen, H.; Zhang, C.; Li, J.; Zhao, M.; Lei, C.; Zhang, A.; Zhang, L. An AluI PCR-RFLP detecting a silent allele at the goat POU1F1 locus and its association with production traits. *Small Ruminant Research* **2007**, *73*, 8-12.
21. Li, S.; Crenshaw, E.B.; Rawson, E.J.; Simmons, D.M.; Swanson, L.W.; Rosenfeld, M.G. Dwarf locus mutants lacking three pituitary cell types result from mutations in the POU-domain gene pit-1. *Nature* **1990**, *347*, 528.
22. Parton, R.; Way, M.; Stang, E. Caveolin-3 associates with developing T-tubules during muscle differentiation: Evidence for a role for caveolins in plasma membrane morphogenesis. *Molecular Biology of the Cell* **1996**, *7*, 1963-1963.
23. Le Lay, S.; Kurzchalia, T.V. Getting rid of caveolins: phenotypes of caveolin-deficient animals. *Biochimica et Biophysica Acta (BBA)-Molecular Cell Research* **2005**, *1746*, 322-333.
24. Williams, T.M.; Lisanti, M.P. The Caveolin genes: from cell biology to medicine. *Annals of medicine* **2004**, *36*, 584-595.
25. Singh, U.; Roswall, P.; Uhrbom, L.; Westermark, B. CGGBP1 regulates cell cycle in cancer cells. *BMC molecular biology* **2011**, *12*, 28.
26. Singh, U.; Westermark, B. CGGBP1 is a nuclear and midbody protein regulating abscission. *Experimental cell research* **2011**, *317*, 143-150.
27. Patel, D.; Patel, M.; Westermark, B.; Singh, U. Dynamic bimodal changes in CpG and non-CpG methylation genome-wide upon CGGBP1 loss-of-function. *BMC research notes* **2018**, *11*, 419.
28. Solinas, G.; Summermatter, S.; Mainieri, D.; Gubler, M.; Montani, J.-P.; Seydoux, J.; Smith, S.; Dulloo, A.G. Corticotropin-releasing hormone directly stimulates thermogenesis in skeletal muscle possibly through substrate cycling between de novo lipogenesis and lipid oxidation. *Endocrinology* **2006**, *147*, 31-38.
29. Buchanan, F.; Thue, T.; Yu, P.; Winkelman-Sim, D. Single nucleotide polymorphisms in the corticotrophin-releasing hormone and pro-opiomelancortin genes are associated with growth and carcass yield in beef cattle. *Animal genetics* **2005**, *36*, 127-131.
30. Li, X.; Wu, Z.L.; Gong, Y.; Liu, Y.; Liu, Z.; Wang, X.; Xin, T.; Ji, Q. Single-nucleotide polymorphism identification in the caprine myostatin gene. *Journal of Animal Breeding and Genetics* **2006**, *123*, 141-144.
31. Miranda, M.; Amigues, Y.; Boscher, M.; Ménissier, F.; Cortés, O.; Dunner, S. Simultaneous genotyping to detect myostatin gene polymorphism in beef cattle breeds. *Journal of animal breeding and genetics* **2002**, *119*, 361-366.
32. Jiang, Y.; Li, N.; Fan, X.; Xiao, L.; Xiang, R.; Hu, X.; Du, L.; Wu, C. Associations of T→ A mutation in the promoter region of myostatin gene with birth weight in Yorkshire pigs. *Asian-Australasian Journal of Animal Sciences* **2002**, *15*, 1543-1545.
33. Gu, Z.-L.; Zhang, H.-F.; Zhu, D.-H.; Li, H. Single nucleotide polymorphism analysis of the chicken Myostatin gene in different chicken lines. *Yi chuan xue bao= Acta genetica Sinica* **2002**, *29*, 599-606.
34. Kaminski, S. Bovine kappa-casein (CASK) gene-molecular nature and application in dairy cattle breeding. *Journal of Applied Genetics* **1996**, *2*, 176-196.
35. Barroso, A.; Dunner, S.; Cañón, J. Polimorfismo genético de las lactoproteínas de los rumiantes domésticos-Revisión. *ITEA* **1999**, *2*, 143-179.
36. Bernard, C.; Cassar-Malek, I.; Le Cunff, M.; Dubroeucq, H.; Renand, G.; Hocquette, J.-F. New indicators of beef sensory quality revealed by expression of specific genes. *Journal of Agricultural and Food Chemistry* **2007**, *55*, 5229-5237.
37. Glass, D.J. Skeletal muscle hypertrophy and atrophy signaling pathways. *The international journal of biochemistry & cell biology* **2005**, *37*, 1974-1984.

38. Neyama, H.; Hamada, Y.; Tsukahara, R.; Narita, M.; Tsukamoto, K.; Ueda, H. Blockade of analgesic effects following systemic administration of N-methyl-kyotorphin, NMYR and arginine in mice deficient of preproenkephalin or proopiomelanocortin gene. *Peptides* **2018**, *107*, 10-16.
39. Fang, X.; Xu, H.; Zhang, C.; Chen, H.; Hu, X.; Gao, X.; Gu, C.; Yue, W. Polymorphism in BMP4 gene and its association with growth traits in goats. *Molecular biology reports* **2009**, *36*, 1339-1344.
40. Wozney, J.M.; Rosen, V.; Celeste, A.J.; Mitsock, L.M.; Whitters, M.J.; Kriz, R.W.; Hewick, R.M.; Wang, E.A. Novel regulators of bone formation: molecular clones and activities. *Science* **1988**, *242*, 1528-1534.
41. Malan, S. The improved Boer goat. *Small Ruminant Research* **2000**, *36*, 165-170.
42. Lau, P.; Nixon, S.J.; Parton, R.G.; Muscat, G.E. ROR α regulates the expression of genes involved in lipid homeostasis in skeletal muscle cells: Caveolin-3 and CPT-1 are direct targets of ROR. *Journal of Biological Chemistry* **2004**.
43. Silveira, A.C.; Morrison, M.A.; Ji, F.; Xu, H.; Reinecke, J.B.; Adams, S.M.; Arneberg, T.M.; Janssian, M.; Lee, J.-E.; Yuan, Y. Convergence of linkage, gene expression and association data demonstrates the influence of the RAR-related orphan receptor alpha (RORA) gene on neovascular AMD: a systems biology based approach. *Vision research* **2010**, *50*, 698-715.
44. Sevane, N.; Armstrong, E.; Wiener, P.; Wong, R.P.; Dunner, S.; Consortium, G. Polymorphisms in twelve candidate genes are associated with growth, muscle lipid profile and meat quality traits in eleven European cattle breeds. *Molecular biology reports* **2014**, *41*, 4721-4731.
45. Sevane, N.; Crespo, I.; Cañón, J.; Dunner, S. A Primer-Extension Assay for simultaneous use in cattle Genotype Assisted Selection, parentage and traceability analysis. *Livestock Science* **2011**, *137*, 141-150.
46. Thaller, G.; Kühn, C.; Winter, A.; Ewald, G.; Bellmann, O.; Wegner, J.; Zühlke, H.; Fries, R. DGAT1, a new positional and functional candidate gene for intramuscular fat deposition in cattle. *Animal genetics* **2003**, *34*, 354-357.
47. Huang, D.; Wang, J.; Liu, Q.; Chu, M.; Di, R.; He, J.; Cao, G.; Fang, L.; Feng, T.; Li, N. Analysis on DNA sequence of TSHB gene and its association with reproductive seasonality in goats. *Molecular biology reports* **2013**, *40*, 1893-1904.
48. Pan, Y.; Zhang, L.; Liu, Q.; Li, Y.; Guo, H.; Peng, Y.; Peng, H.; Tang, B.; Hu, Z.; Zhao, J. Insertion of a knockout-first cassette in Ampd1 gene leads to neonatal death by disruption of neighboring genes expression. *Scientific reports* **2016**, *6*, 35970.
49. Zhang, L.; Michal, J.J.; O'Fallon, J.V.; Pan, Z.; Gaskins, C.T.; Reeves, J.J.; Busboom, J.R.; Zhou, X.; Ding, B.; Dodson, M.V. Quantitative genomics of 30 complex phenotypes in Wagyu x Angus F1 progeny. *International journal of biological sciences* **2012**, *8*, 838.
50. Kırıkçı, K.; Noce, A.; Zidi, A.; Serradilla, J.M.; Carrizosa, J.; Urrutia, B.; Pilla, F.; D'Andrea, M.; Capote, J.; Bizelis, I. Analysing the diversity of the caprine melanocortin 1 receptor (MC1R) in goats with distinct geographic origins. *Small ruminant research* **2016**, *145*, 7-11.
51. Ropka-Molik, K.; Żukowski, K.; Eckert, R.; Piórkowska, K.; Oczkowicz, M.; Gurgul, A.; Szmatoła, T. Whole transcriptome analysis of the porcine muscle tissue of breeds differing in muscularity and meat quality traits. *Livestock Science* **2015**, *182*, 93-100.
52. Lindholm-Perry, A.; Kuehn, L.; Smith, T.; Ferrell, C.; Jenkins, T.; Freetly, H.; Snelling, W. A region on BTA14 that includes the positional candidate genes LYPLA1, XKR4 and TMEM68 is associated with feed intake and growth phenotypes in cattle 1. *Animal genetics* **2012**, *43*, 216-219.
53. Rubin, C.-J.; Megens, H.-J.; Barrio, A.M.; Maqbool, K.; Sayyab, S.; Schwochow, D.; Wang, C.; Carlborg, Ö.; Jern, P.; Jørgensen, C.B. Strong signatures of selection in the domestic pig genome. *Proceedings of the National Academy of Sciences* **2012**, *109*, 19529-19536.
54. Pryce, J.E.; Hayes, B.J.; Bolormaa, S.; Goddard, M.E. Polymorphic regions affecting human height also control stature in cattle. *Genetics* **2011**, *187*, 981-984.
55. Hughes, A.L.; Nei, M. Pattern of nucleotide substitution at major histocompatibility complex class I loci reveals overdominant selection. *Nature* **1988**, *335*, 167.

56. Kemper, K.E.; Saxton, S.J.; Bolormaa, S.; Hayes, B.J.; Goddard, M.E. Selection for complex traits leaves little or no classic signatures of selection. *BMC genomics* **2014**, *15*, 246.
57. Petersen, J.L.; Mickelson, J.R.; Rendahl, A.K.; Valberg, S.J.; Andersson, L.S.; Axelsson, J.; Bailey, E.; Bannasch, D.; Binns, M.M.; Borges, A.S. Genome-wide analysis reveals selection for important traits in domestic horse breeds. *PLoS genetics* **2013**, *9*, e1003211.
58. Bellinge, R.; Liberles, D.A.; Iaschi, S.; O'brien, P.; Tay, G. Myostatin and its implications on animal breeding: a review. *Animal genetics* **2005**, *36*, 1-6.
59. Flori, L.; Fritz, S.; Jaffrézic, F.; Boussaha, M.; Gut, I.; Heath, S.; Foulley, J.-L.; Gautier, M. The genome response to artificial selection: a case study in dairy cattle. *PloS one* **2009**, *4*, e6595.
60. Sharma, A.; Lee, J.S.; Dang, C.G.; Sudrajad, P.; Kim, H.C.; Yeon, S.H.; Kang, H.S.; Lee, S.-H. Stories and challenges of genome wide association studies in livestock—A review. *Asian-Australasian journal of animal sciences* **2015**, *28*, 1371.
61. Kijas, J.W.; Lenstra, J.A.; Hayes, B.; Boitard, S.; Neto, L.R.P.; San Cristobal, M.; Servin, B.; McCulloch, R.; Whan, V.; Gietzen, K. Genome-wide analysis of the world's sheep breeds reveals high levels of historic mixture and strong recent selection. *PLoS biology* **2012**, *10*, e1001258.
62. Al-Mamun, H.A.; Kwan, P.; Clark, S.A.; Ferdosi, M.H.; Tellam, R.; Gondro, C. Genome-wide association study of body weight in Australian Merino sheep reveals an orthologous region on OAR6 to human and bovine genomic regions affecting height and weight. *Genetics Selection Evolution* **2015**, *47*, 66.
63. Cao, J.; Wei, C.; Liu, D.; Wang, H.; Wu, M.; Xie, Z.; Capellini, T.D.; Zhang, L.; Zhao, F.; Li, L. DNA methylation Landscape of body size variation in sheep. *Scientific reports* **2015**, *5*, 13950.
64. Guan, D.; Luo, N.; Tan, X.; Zhao, Z.; Huang, Y.; Na, R.; Zhang, J.; Zhao, Y. Scanning of selection signature provides a glimpse into important economic traits in goats (Capra hircus). *Scientific Reports* **2016**, *6*, 36372.
65. Fijneman, R.J.; Bade, L.K.; Peham, J.R.; Van De Wiel, M.A.; Van Hinsbergh, V.W.; Meijer, G.A.; O'Sullivan, M.G.; Cormier, R.T. Pla2g2a attenuates colon tumorigenesis in azoxymethane-treated C57BL/6 mice; expression studies reveal Pla2g2a target genes and pathways. *Analytical Cellular Pathology* **2009**, *31*, 345-356.
66. Kahyo, T.; Iwaizumi, M.; Shinmura, K.; Matsuura, S.; Nakamura, T.; Watanabe, Y.; Yamada, H.; Sugimura, H. A novel tumor-derived SGOL1 variant causes abnormal mitosis and unstable chromatid cohesion. *Oncogene* **2011**, *30*, 4453.
67. Huppke, P.; Brendel, C.; Kalscheuer, V.; Korenke, G.C.; Marquardt, I.; Freisinger, P.; Christodoulou, J.; Hillebrand, M.; Pitelet, G.; Wilson, C. Mutations in SLC33A1 cause a lethal autosomal-recessive disorder with congenital cataracts, hearing loss, and low serum copper and ceruloplasmin. *The American Journal of Human Genetics* **2012**, *90*, 61-68.
68. Wu, L.; Wary, K.K.; Revskoy, S.; Gao, X.; Tsang, K.; Komarova, Y.A.; Rehman, J.; Malik, A.B. Histone demethylases KDM4A and KDM4C regulate differentiation of embryonic stem cells to endothelial cells. *Stem cell reports* **2015**, *5*, 10-21.
69. Tong, G.Q.; Heng, B.C.; Tan, L.G.; Ng, S.C. Aberrant profile of gene expression in cloned mouse embryos derived from donor cumulus nuclei. *Cell and tissue research* **2006**, *325*, 231-243.
70. Carlsson, P.; Mahlapuu, M. Forkhead transcription factors: key players in development and metabolism. *Developmental biology* **2002**, *250*, 1-23.
71. Uhlenhaut, N.H.; Treier, M. Foxl2 function in ovarian development. *Molecular genetics and metabolism* **2006**, *88*, 225-234.
72. Vaiman, D.; Koutita, O.; Oustry, A.; Elsen, J.-M.; Manfredi, E.; Fellous, M.; Cribiu, E. Genetic mapping of the autosomal region involved in XX sex-reversal and horn development in goats. *Mammalian Genome* **1996**, *7*, 133-137.
73. Messer, L.A.; Wang, L.; Tuggle, C.K.; Yerle, M.; Chardon, P.; Pomp, D.; Womack, J.E.; Barendse, W.; Crawford, A.M.; Notter, D.R. Mapping of the melatonin receptor la (MTNR1A) gene in pigs, sheep, and cattle. *Mammalian genome* **1997**, *8*, 368-370.

74. Chu, M.; He, Y.; Cheng, D.; Ye, S.; Fang, L.; Wang, J. Association between expression of reproductive seasonality and alleles of melatonin receptor 1A in goats. *Animal reproduction science* **2007**, *101*, 276-284.
75. Migaud, M.; Gavet, S.; Pelletier, J. Partial cloning and polymorphism of the melatonin~ 1~ a (Mel~ 1~ a) receptor gene in two breeds of goat with different reproductive seasonality. *REPRODUCTION-CAMBRIDGE-* **2002**, *124*, 59-64.
76. Shi, L.; Yue, W.; Ren, Y.; Lei, F.; Zhao, J. Sex determination in goat by amplification of the HMG box using duplex PCR. *Animal reproduction science* **2008**, *105*, 398-403.
77. Sullivan, K.M.; Mannucci, A.; Kimpton, C.P.; Gill, P. A rapid and quantitative DNA sex test: fluorescence-based PCR analysis of XY homologous gene amelogenin. *Biotechniques* **1993**, *15*, 636-638, 640-631.
78. Chen, C.; Hu, C.; Wang, C.; Hung, C.; Wu, H.; Choo, K.; Cheng, W. Gender determination in single bovine blastomeres by polymerase chain reaction amplification of sex-specific polymorphic fragments in the amelogenin gene. *Molecular Reproduction and Development: Incorporating Gamete Research* **1999**, *54*, 209-214.
79. Chang, Z.; Fan, X.; Luo, M.; Wu, Z.; Tan, J. Factors affecting superovulation and embryo transfer in Boer goats. *Asian-australasian journal of animal sciences* **2006**, *19*, 341-346.
80. Chen, A.-q.; Xu, Z.-r.; Yu, S.-d. Sexing goat embryos by PCR amplification of X-and Y-chromosome specific sequence of the Amelogenin gene. *Asian-Australasian Journal of Animal Sciences* **2007**, *20*, 1689-1693.
81. Cui, Y.; Yan, H.; Wang, K.; Xu, H.; Zhang, X.; Zhu, H.; Liu, J.; Qu, L.; Lan, X.; Pan, C. Insertion/deletion within the KDM6A gene is significantly associated with litter size in goat. *Frontiers in genetics* **2018**, *9*, 91.
82. Saleh, A., Abdelkader. Utilization of molecular markers to detect some genes and mutations affecting economic traits in Egyptian sheep breeds. *M.Sc Thesis Alexandria. University* **2016**.
83. Bemji, M.; Isa, A.; Ibeagha-Awemu, E.; Wheto, M. Polymorphisms of caprine GnRHR gene and their association with litter size in West African Dwarf goats. *Molecular biology reports* **2018**, *45*, 63-69.
84. Podzus, J.; Kowalczyk-Quintas, C.; Schuepbach-Mallepell, S.; Willen, L.; Staehlin, G.; Vigolo, M.; Tardivel, A.; Headon, D.; Kirby, N.; Mikkola, M.L. Ectodysplasin A in biological fluids and diagnosis of ectodermal dysplasia. *Journal of dental research* **2017**, *96*, 217-224.
85. Carneiro, M.; Hu, D.; Archer, J.; Feng, C.; Afonso, S.; Chen, C.; Blanco-Aguiar, J.A.; Garreau, H.; Boucher, S.; Ferreira, P.G. Dwarfism and altered craniofacial development in rabbits is caused by a 12.1 kb deletion at the HMGA2 locus. *Genetics* **2017**, *205*, 955-965.
86. Chung, J.; Zhang, X.; Collins, B.; Sper, R.B.; Gleason, K.; Simpson, S.; Koh, S.; Sommer, J.; Flowers, W.L.; Petters, R.M. High mobility group A2 (HMGA2) deficiency in pigs leads to dwarfism, abnormal fetal resource allocation, and cryptorchidism. *Proceedings of the National Academy of Sciences* **2018**, *115*, 5420-5425.
87. Zhao, H.; Li, Z.; Cooney, A.J.; Lan, Z.-J. Orphan nuclear receptor function in the ovary. *Front Biosci* **2007**, *12*, 405.
88. Wang, Q.; Cooney, A.J. Revisiting the role of GCNF in embryonic development. In Proceedings of the Seminars in cell & developmental biology, 2013; pp. 679-686.
89. He, B.; Mi, Y.; Zhang, C. Gonadotropins regulate ovarian germ cell mitosis/meiosis decision in the embryonic chicken. *Molecular and cellular endocrinology* **2013**, *370*, 32-41.
90. Młodawska, W.; Grzesiak, M.; Kochan, J.; Nowak, A. Intrafollicular level of steroid hormones and the expression of androgen receptor in the equine ovary at puberty. *Theriogenology* **2018**, *121*, 13-20.
91. de Santi, F.; Beltrame, F.L.; Hinton, B.T.; Cerri, P.S.; Sasso-Cerri, E. Reduced levels of stromal sex hormone-binding globulin and androgen receptor dysfunction in the sperm storage region of the rat epididymis. *Reproduction* **2018**, *155*, 467-479.

92. O'Hara, L.; Smith, L.B. Androgen receptor roles in spermatogenesis and infertility. *Best Practice & Research Clinical Endocrinology & Metabolism* **2015**, *29*, 595-605.
93. Celik, O.; Celik, N.; Gungor, S.; Haberal, E.T.; Aydin, S. Selective regulation of oocyte meiotic events enhances progress in fertility preservation methods. *Biochemistry insights* **2015**, *8*, BCI. S28596.
94. Tu, X.; Liu, M.; Tang, J.; Zhang, Y.; Shi, Y.; Yu, L.; Sun, Z. The ovarian estrogen synthesis function was impaired in Y123F mouse and partly restored by exogenous FSH supplement. *Reproductive Biology and Endocrinology* **2018**, *16*, 44.
95. Chen, C.; Jiang, X.; Li, Y.; Yu, H.; Li, S.; Zhang, Z.; Xu, H.; Yang, Y.; Liu, G.; Zhu, F. Low-dose oral copper treatment changes the hippocampal phosphoproteomic profile and perturbs mitochondrial function in a mouse model of Alzheimer's disease. *Free Radical Biology and Medicine* **2019**.
96. Myers, C.T.; Stong, N.; Mountier, E.I.; Helbig, K.L.; Freytag, S.; Sullivan, J.E.; Zeev, B.B.; Nissenkorn, A.; Tzadok, M.; Heimer, G. De novo mutations in PPP3CA cause severe neurodevelopmental disease with seizures. *The American Journal of Human Genetics* **2017**, *101*, 516-524.
97. Saleh, A.A.; Rashad, A.M.; Hassanine, N.N.; Sharaby, M.A.; Zhao, Y. Comparative analysis of IGFBP-3 gene sequence in Egyptian sheep, cattle, and buffalo. *BMC research notes* **2019**, *12*, 623.
98. Zhang, Q.; Calus, M.P.; Guldbrandtsen, B.; Lund, M.S.; Sahana, G. Estimation of inbreeding using pedigree, 50k SNP chip genotypes and full sequence data in three cattle breeds. *BMC genetics* **2015**, *16*, 88.
99. Stella, A.; Ajmone-Marsan, P.; Lazzari, B.; Boettcher, P. Identification of selection signatures in cattle breeds selected for dairy production. *Genetics* **2010**, *185*, 1451-1461.
100. Supakorn, C. The important candidate genes in goats—a review. *Walailak J. Sci. Tech* **2009**, *6*, 17-36.
101. Maj, A.; Korczak, M.; Bagnicka, E.; Zwierzchowski, L.; Pierzchała, M. A TG-repeat polymorphism in the 5′-noncoding region of the goat growth hormone receptor gene and search for its association with milk production traits. *Small ruminant research* **2007**, *67*, 279-284.
102. Grosclaude, F.; Mahé, M.-F.; Brignon, G.; Di Stasio, L.; Jeunet, R. A Mendelian polymorphism underlying quantitative variations of goat α s1-casein. *Genetique, selection, evolution* **1987**, *19*, 399.
103. Sacchi, P.; Chessa, S.; Budelli, E.; Bolla, P.; Ceriotti, G.; Soglia, D.; Rasero, R.; Cauvin, E.; Caroli, A. Casein haplotype structure in five Italian goat breeds. *Journal of Dairy Science* **2005**, *88*, 1561-1568.
104. Sztankoova, Z.; Senese, C.; Czernekova, V.; Dudkova, G.; Kott, T.; Matlova, V.; Soldat, J. Genomic analysis of the CSN2 and CSN3 loci in two Czech goat breeds. *Animal Science Papers and Reports* **2005**, *23*, 67-70.
105. Rijnkels, M. Multispecies comparison of the casein gene loci and evolution of casein gene family. *Journal of mammary gland biology and neoplasia* **2002**, *7*, 327-345.
106. Cosenza, G.; Pauciullo, A.; Colimoro, L.; Mancusi, A.; Rando, A.; Di Berardino, D.; Ramunno, L. An SNP in the goat CSN2 promoter region is associated with the absence of β-casein in milk. *Animal genetics* **2007**, *38*, 655-658.
107. Fariello, M.-I.; Servin, B.; Tosser-Klopp, G.; Rupp, R.; Moreno, C.; San Cristobal, M.; Boitard, S.; Consortium, I.S.G. Selection signatures in worldwide sheep populations. *PLoS One* **2014**, *9*, e103813.
108. Fortes, M.; Lehnert, S.; Bolormaa, S.; Reich, C.; Fordyce, G.; Corbet, N.; Whan, V.; Hawken, R.; Reverter, A. Finding genes for economically important traits: Brahman cattle puberty. *Animal Production Science* **2012**, *52*, 143-150.
109. Martin, P.; Palhière, I.; Maroteau, C.; Bardou, P.; Canale-Tabet, K.; Sarry, J.; Woloszyn, F.; Bertrand-Michel, J.; Racke, I.; Besir, H. A genome scan for milk production traits in dairy goats reveals two new mutations in Dgat1 reducing milk fat content. *Scientific reports* **2017**, *7*, 1872.

110. Bertolini, F.; Servin, B.; Talenti, A.; Rochat, E.; Kim, E.S.; Oget, C.; Palhière, I.; Crisà, A.; Catillo, G.; Steri, R. Signatures of selection and environmental adaptation across the goat genome post-domestication. *Genetics Selection Evolution* **2018**, *50*, 57.
111. Salo, A.M.; Cox, H.; Farndon, P.; Moss, C.; Grindulis, H.; Risteli, M.; Robins, S.P.; Myllylä, R. A connective tissue disorder caused by mutations of the lysyl hydroxylase 3 gene. *The American Journal of Human Genetics* **2008**, *83*, 495-503.
112. Sansregret, L.; Nepveu, A. The multiple roles of CUX1: insights from mouse models and cell-based assays. *Gene* **2008**, *412*, 84-94.
113. Rao, C.; Foernzler, D.; Loftus, S.K.; Liu, S.; McPherson, J.D.; Jungers, K.A.; Apte, S.S.; Pavan, W.J.; Beier, D.R. A defect in a novel ADAMTS family member is the cause of the belted white-spotting mutation. *Development* **2003**, *130*, 4665-4672.
114. Hosking, B.M.; Wang, S.M.; Chen, S.L.; Penning, S.; Koopman, P.; Muscat, G.E. SOX18 directly interacts with MEF2C in endothelial cells. *Biochemical and biophysical research communications* **2001**, *287*, 493-500.
115. Badaoui, B.; Manunza, A.; Castelló, A.; D'Andrea, M.; Pilla, F.; Capote, J.; Jordana, J.; Ferrando, A.; Martínez, A.; Cabrera, B. Advantages and limitations of authenticating Palmera goat dairy products by pyrosequencing the melanocortin 1 receptor (MC1R) gene. *Journal of dairy science* **2014**, *97*, 7293-7297.
116. Hayes, B.J.; Pryce, J.; Chamberlain, A.J.; Bowman, P.J.; Goddard, M.E. Genetic architecture of complex traits and accuracy of genomic prediction: coat colour, milk-fat percentage, and type in Holstein cattle as contrasting model traits. *PLoS Genetics* **2010**, *6*, e1001139.
117. Sulem, P.; Gudbjartsson, D.F.; Stacey, S.N.; Helgason, A.; Rafnar, T.; Magnusson, K.P.; Manolescu, A.; Karason, A.; Palsson, A.; Thorleifsson, G. Genetic determinants of hair, eye and skin pigmentation in Europeans. *Nature genetics* **2007**, *39*, 1443.
118. Han, J.; Kraft, P.; Nan, H.; Guo, Q.; Chen, C.; Qureshi, A.; Hankinson, S.E.; Hu, F.B.; Duffy, D.L.; Zhao, Z.Z. A genome-wide association study identifies novel alleles associated with hair color and skin pigmentation. *PLoS genetics* **2008**, *4*, e1000074.
119. Guo, J.; Tao, H.; Li, P.; Li, L.; Zhong, T.; Wang, L.; Ma, J.; Chen, X.; Song, T.; Zhang, H. Whole-genome sequencing reveals selection signatures associated with important traits in six goat breeds. *Scientific reports* **2018**, *8*, 10405.
120. Sundram, U.; Harvell, J.D.; Rouse, R.V.; Natkunam, Y. Expression of the B-cell proliferation marker MUM1 by melanocytic lesions and comparison with S100, gp100 (HMB45), and MelanA. *Modern pathology* **2003**, *16*, 802.
121. Hernandez, D.G.; Mucha, S.; Banos, G.; Kaseja, K.; Moore, K.; Lambe, N.; Yates, J.; Bunger, L. Analysis of single nucleotide polymorphisms variation associated with important economic and computed tomography measured traits in Texel sheep. *animal* **2018**, *12*, 915-922.
122. Armstrong, E.; Ciappesoni, G.; Iriarte, W.; Da Silva, C.; Macedo, F.; Navajas, E.; Brito, G.; San Julián, R.; Gimeno, D.; Postiglioni, A. Novel genetic polymorphisms associated with carcass traits in grazing Texel sheep. *Meat science* **2018**, *145*, 202-208.
123. Casas, E.; Shackelford, S.; Keele, J.; Koohmaraie, M.; Smith, T.; Stone, R. Detection of quantitative trait loci for growth and carcass composition in cattle. *Journal of animal science* **2003**, *81*, 2976-2983.
124. Gutiérrez-Gil, B.; Williams, J.; Homer, D.; Burton, D.; Haley, C.; Wiener, P. Search for quantitative trait loci affecting growth and carcass traits in a cross population of beef and dairy cattle. *Journal of animal science* **2009**, *87*, 24-36.
125. Bolormaa, S.; Hayes, B.J.; van der Werf, J.H.; Pethick, D.; Goddard, M.E.; Daetwyler, H.D. Detailed phenotyping identifies genes with pleiotropic effects on body composition. *BMC genomics* **2016**, *17*, 1-21.
126. Matika, O.; Riggio, V.; Anselme-Moizan, M.; Law, A.S.; Pong-Wong, R.; Archibald, A.L.; Bishop, S.C. Genome-wide association reveals QTL for growth, bone and in vivo carcass traits as assessed by computed tomography in Scottish Blackface lambs. *Genetics Selection Evolution* **2016**, *48*, 11.

127. Marrube, G.; Cano, E.; Roldán, D.L.; Bidinost, F.; Abad, M.; Allain, D.; Vaiman, D.; Taddeo, H.; Poli, M.A. QTL affecting conformation traits in Angora goats. *Small ruminant research* **2007**, *71*, 255-263.
128. Kominakis, A.; Hager-Theodorides, A.L.; Zoidis, E.; Saridaki, A.; Antonakos, G.; Tsiamis, G. Combined GWAS and 'guilt by association'-based prioritization analysis identifies functional candidate genes for body size in sheep. *Genetics Selection Evolution* **2017**, *49*, 41.
129. Detection of copy number variations and their effects in Chinese bulls. *BMC Genomics* **2014**, *15*, 480, doi:DOI: 10.1186/1471-2164-15-480
130. Hiendleder, S.; Thomsen, H.; Reinsch, N.; Bennewitz, J.; Leyhe-Horn, B.; Looft, C.; Xu, N.; Medjugorac, I.; Russ, I.; Kühn, C. Mapping of QTL for body conformation and behavior in cattle. *Journal of Heredity* **2003**, *94*, 496-506.
131. Visser, C.; Van Marle-Köster, E.; Snyman, M.; Bovenhuis, H.; Crooijmans, R. Quantitative trait loci associated with pre-weaning growth in South African Angora goats. *Small Ruminant Research* **2013**, *112*, 15-20.
132. Riggio, V.; Matika, O.; Pong-Wong, R.; Stear, M.; Bishop, S. Genome-wide association and regional heritability mapping to identify loci underlying variation in nematode resistance and body weight in Scottish Blackface lambs. *Heredity* **2013**, *110*, 420-429.
133. Zhang, L.; Liu, J.; Zhao, F.; Ren, H.; Xu, L.; Lu, J.; Zhang, S.; Zhang, X.; Wei, C.; Lu, G. Genome-wide association studies for growth and meat production traits in sheep. *PloS one* **2013**, *8*, e66569.
134. Jalil-Sarghale, A.; Shahrbabak, M.M.; Sharbabak, H.M.; Sadeghi, M.; Mura, M. Association of pituitary specific transcription factor-1 (POU1F1) gene polymorphism with growth and biometric traits and blood metabolites in Iranian Zel and Lori-Bakhtiari sheep. *Molecular biology reports* **2014**, *41*, 5787-5792.
135. Abadi, M.M.; Askari, N.; Baghizadeh, A.; Esmailizadeh, A. A directed search around caprine candidate loci provided evidence for microsatellites linkage to growth and cashmere yield in Rayini goats. *Small Ruminant Research* **2009**, *81*, 146-151.
136. Miyata, M.; Gasparin, G.; Coutinho, L.L.; Martinez, M.L.; Machado, M.A.; Silva, M.V.G.; Campos, A.L.; Sonstegard, T.S.; Rosário, M.F.d.; Regitano, L.C.d.A. Quantitative trait loci (QTL) mapping for growth traits on bovine chromosome 14. *Genetics and Molecular Biology* **2007**, *30*, 364-369.
137. Cano, E.; Marrube, G.; Roldán, D.L.; Bidinost, F.; Abad, M.; Allain, D.; Vaiman, D.; Taddeo, H.; Poli, M.A. QTL affecting fleece traits in Angora goats. *Small Ruminant Research* **2007**, *71*, 158-164.
138. Visser, C.; Van Marle-Köster, E.; Bovenhuis, H.; Crooijmans, R. QTL for mohair traits in South African Angora goats. *Small ruminant research* **2011**, *100*, 8-14.
139. C. De La Chevrotiere, S.C.B., R. Arquet et al. Detection of quantitative trait loci for resistance to gastrointestinal nematode infections in Creole goats. *Animal Genetics* **2012**, *43. 6*pp. 768–775
140. Bolormaa, S.; Van Der Werf, J.; Walkden-Brown, S.W.; Marshall, K.; Ruvinsky, A. A quantitative trait locus for faecal worm egg and blood eosinophil counts on chromosome 23 in Australian goats. *Journal of Animal Breeding and Genetics* **2010**, *127*, 207-214.
141. Hanotte, O.; Ronin, Y.; Agaba, M.; Nilsson, P.; Gelhaus, A.; Horstmann, R.; Sugimoto, Y.; Kemp, S.; Gibson, J.; Korol, A. Mapping of quantitative trait loci controlling trypanotolerance in a cross of tolerant West African N'Dama and susceptible East African Boran cattle. *Proceedings of the National Academy of Sciences* **2003**, *100*, 7443-7448.
142. Roldán, D.; Rabasa, A.; Saldaño, S.; Holgado, F.; Poli, M.; Cantet, R. QTL detection for milk production traits in goats using a longitudinal model. *Journal of Animal Breeding and Genetics* **2008**, *125*, 187-193.
143. Zhang, Q.; Boichard, D.; Hoeschele, I.; Ernst, C.; Eggen, A.; Murkve, B.; Pfister-Genskow, M.; Witte, L.A.; Grignola, F.E.; Uimari, P. Mapping quantitative trait loci for milk production and health of dairy cattle in a large outbred pedigree. *Genetics* **1998**, *149*, 1959-1973.

Supplementary information file (2); Supplementary Tables
